# Evaluation of a transdiagnostic psychodynamic online intervention to support return to work: A randomized controlled trial

**DOI:** 10.1371/journal.pone.0176513

**Published:** 2017-05-08

**Authors:** Rüdiger Zwerenz, Jan Becker, Katharina Gerzymisch, Martin Siepmann, Martin Holme, Ulrich Kiwus, Sieglinde Spörl-Dönch, Manfred E. Beutel

**Affiliations:** 1 Department of Psychosomatic Medicine and Psychotherapy, University Medical Center of the Johannes Gutenberg-University Mainz, Mainz, Germany; 2 University Medical Center of the Carl Gustav Carus Technical University, Department of Psychosomatic Medicine and Psychotherapy, Dresden, Germany; 3 German Statutory Pension Insurance Rehabilitation Center for Orthopedic Diseases, Clinic Weser, Bad Pyrmont, Germany; 4 German Statutory Pension Insurance Rehabilitation Center for Cardiovascular Diseases, Clinic Wetterau, Bad Nauheim, Germany; 5 Clinic for Prevention and Rehabilitation of Cardiovascular Diseases ‘Haus Franken’ GmbH, Bad Neustadt/ Saale, Germany; Clinical Psychology, GERMANY

## Abstract

**Objectives:**

Given their flexibility, online interventions may be useful as an outpatient treatment option to support vocational reintegration after inpatient rehabilitation. To that purpose we devised a transdiagnostic psychodynamic online intervention to facilitate return to work, focusing on interpersonal conflicts at the workplace often responsible for work-related stress.

**Research design and methods:**

In a randomized controlled trial, we included employed patients from cardiologic, psychosomatic and orthopedic rehabilitation with work-related stress or need for support at intake to inpatient rehabilitation after they had given written consent to take part in the study. Following discharge, maladaptive interpersonal interactions at the workplace were identified via weekly blogs and processed by written therapeutic comments over 12 weeks in the intervention group (IG). The control group (CG) received an augmented treatment as usual condition. The main outcome, subjective prognosis of gainful employment (SPE), and secondary outcomes (psychological complaints) were assessed by means of online questionnaires before, at the end of aftercare (3 months) and at follow-up (12 months). We used ITT analyses controlling for baseline scores and medical group.

**Results:**

*N* = 319 patients were enrolled into IG and *N =* 345 into CG. 77% of the IG logged in to the webpage (CG 74%) and 65% of the IG wrote blogs. Compared to the CG, the IG reported a significantly more positive SPE at follow-up. Measures of depression, anxiety and psychosocial stressors decreased from baseline to follow-up, whereas the corresponding scores increased in the CG. Correspondingly, somatization and psychological quality of life improved in the IG.

**Conclusions:**

Psychodynamic online aftercare was effective to enhance subjective prognosis of future employment and improved psychological complaints across a variety of chronic physical and psychological conditions, albeit with small effect sizes.

## Introduction

Sick leave and premature pension are rising due to common mental disorders such as depression and anxiety disorders which have become the leading cause of sickness absence in the developed countries [1]. On the other hand, chronic work strains, often resulting from rapidly changing work demands, have been identified as important determinants of physical and mental health [2]. In a meta-analysis [3] psychosocial work stressors such as high emotional work demands, low decision latitude and low social support predicted common mental diseases, amplified by individual vulnerability factors such as over-commitment.

In Germany, medical rehabilitation aims at restoring and maintaining work ability [4]. Approximately one third of the German population has reported significant work-related stress [5]. Inpatients of psychosomatic rehabilitation reported not only higher stress, but also fewer resources to cope with work stress when compared to the general German population [6]. Therefore, various work-related interventions have been adopted during inpatient rehabilitation treatment in order to improve vocational reintegration [7]. In previous studies we could show that vocational training during inpatient psychosomatic rehabilitation could improve return to work in the long run [8]. A brief inpatient intervention to identify and reduce vocational stress improved work-related attitudes [9, 10]. However, applying psychological and practical strategies acquired in rehabilitation into daily work and social life remains a critical obstacle for many patients with chronic mental or physical disorders, particularly if they have already had extended leaves of absence from work and if they fail to receive appropriate aftercare supporting return to work.

Currently few patients take part in existing outpatient-treatments following inpatient rehabilitation because of incompatibility with their duties at work / within their families, or poor access to the outpatient rehabilitation facility [11, 12]. For these reasons internet-based interventions seem to be promising, because most people (80% in 2015) in Germany [13] are online and more than half (51.1%) of the German population uses the internet for health related issues [14].

There has been growing evidence that internet-based cognitive behavior therapy improves depression, anxiety and other mental disorders [15, 16]. In the field of psychosomatic rehabilitation, a recent German trial [17] found that participants of online aftercare were able to maintain mental health benefits during the follow-up period, whereas the control group receiving treatment as usual deteriorated significantly. Up to now only few online interventions with a focus on work-related stress exist, and they have been predominantly founded on cognitive behavioral interventions [18, 19]. Current online interventions in this field focus on workplace stress, e.g. with problem solving training [18] or recovery training for sleepless employees with work related stress [19]. Online interventions focusing directly on workplace integration after inpatient stays are still missing but are already integrated in the current definition of ‘occupational e-mental health’, which describes ‘the use of information and communication technology to deliver psychoeducation, health risk assessment, work-place health promotion, preventive interventions (universal, selected, or indicated), treatment, relapse prevention, and return-to-work assistance for the mental health of workers […].’ [20] (p. 259).

To our knowledge, psychodynamic online interventions have been rarely developed and evaluated to date [21]. Recently, Johansson et al. [22, 23] demonstrated the efficacy of an internet-based psychodynamic guided self-help program for generalized anxiety and depressive disorders. Participants completed reading and writing tasks on accessing and confronting emotions with minimal written therapist support providing clarification, encouragement and gradual administration of self-study material.

In order to promote successful vocational reintegration after inpatient medical rehabilitation for people with chronic diseases (psychosomatic, cardiological, orthopedic), we devised a transdiagnostic psycho-educative online aftercare program [24] based on a psychodynamic concept and focusing on interpersonal conflicts at the workplace, often responsible for work-related stress. As we aimed to promote long-term work ability, we used the subjective prognosis of gainful employment as our primary outcome and we used depression, anxiety, psychosocial stressors and quality of life as secondary outcomes. Our hypothesis was that participation at the psychodynamic internet-based aftercare will improve a) subjective prognosis of gainful employment as well as b) comorbid psychological symptoms compared to the usual course after inpatient medical rehabilitation.

## Materials and methods

### Study design

The effectiveness of the psychodynamic internet-based aftercare program was evaluated in a multicenter randomized controlled trial with inpatients from a total of four rehabilitation clinics: one psychosomatic, one orthopedic and two clinics for cardiology. Participants in the intervention group (IG) received the internet-based aftercare intervention, based on a manualized, educational vocational training they attended during inpatient rehabilitation. The control group (CG) also attended the inpatient training and did not get the special internet-based aftercare program, but obtained access to a placebo internet-based program (with links to publicly accessible information about stress management and coping).

Data and outcome measures were gathered before (screening for eligibility), after study intake (T0), at the end of rehabilitation (baseline for aftercare, T1), three months after discharge (end of online intervention, T2) and 12 months after inpatient medical rehabilitation (follow-up, T3) by standardized self-report questionnaires. Patients were recruited between July 1, 2011 and March 31, 2013; the last follow-up (T3) was completed on May 26, 2014.

All patients were instructed to use pseudonyms to login and for all actions on the internet platform so that no personal data were stored on the webserver, rendering user identification impossible. Administration of the internet platform, allocation of the weekly writing tasks and therapeutic feedback were managed by staff of the Department of Psychosomatic Medicine and Psychotherapy of the University Medial Centre of the Johannes Gutenberg University of Mainz. The inpatient stress-management training was conducted by experienced therapists (psychologists or social-workers) of the four rehabilitation clinics.

The Study Centre of Mental Disorders at the University Medical Center was responsible for storing personal data, encoding the participants and randomizing the groups (cluster-randomization). Randomization was done as a block randomization with ‘Research Randomiser’ [25]. Patients were allocated to training groups after screening. Each group was randomized as a cluster within the clinics. Cluster size varied between 2 and 12 participants. Each clinic was assigned 30 blocks, each containing IG and CG in random order and in a 1:1 ratio. Data security was guaranteed by secure sockets layer coded internet connections as used in bank transfers and a firewall-protected webserver for both the MySQL database and the application of the internet platform.

The study was powered to identify small effect sizes (d = .30), with a statistical power of 0.80 and alpha of 0.05. The power was calculated for analysis of covariance (with baseline score as a covariate) and two-sided testing. After correction for cluster randomized trials [26] a minimum sample size of n = 475 was needed for robust statistical analyses. A detailed description of sample size calculation can be found in our published study protocol [24]. The study protocol and the final version of the written informed consent were approved by the Ethics Committee of the Federal State of Rhineland Palatinate (Germany), which is responsible for the Principal Investigator (Ref. No. 837.415.10[7424]; date of approval January 12, 2011) and by the ethics committees responsible for the cooperating rehabilitation clinics.

Due to a tight schedule at the beginning of the study the trial was retrospectively registered (trial registration ISRCTN33957202) six weeks after recruitment of the first patient, however 19 months before the end of recruitment. Furthermore the authors confirm that all ongoing and related trials for this intervention are registered.

The study was funded by the German Statutory Pension Insurance Scheme (Deutsche Rentenversicherung Bund; grant number: 0423/00-40-65-50-25).

### Participants

**Fig 1:**
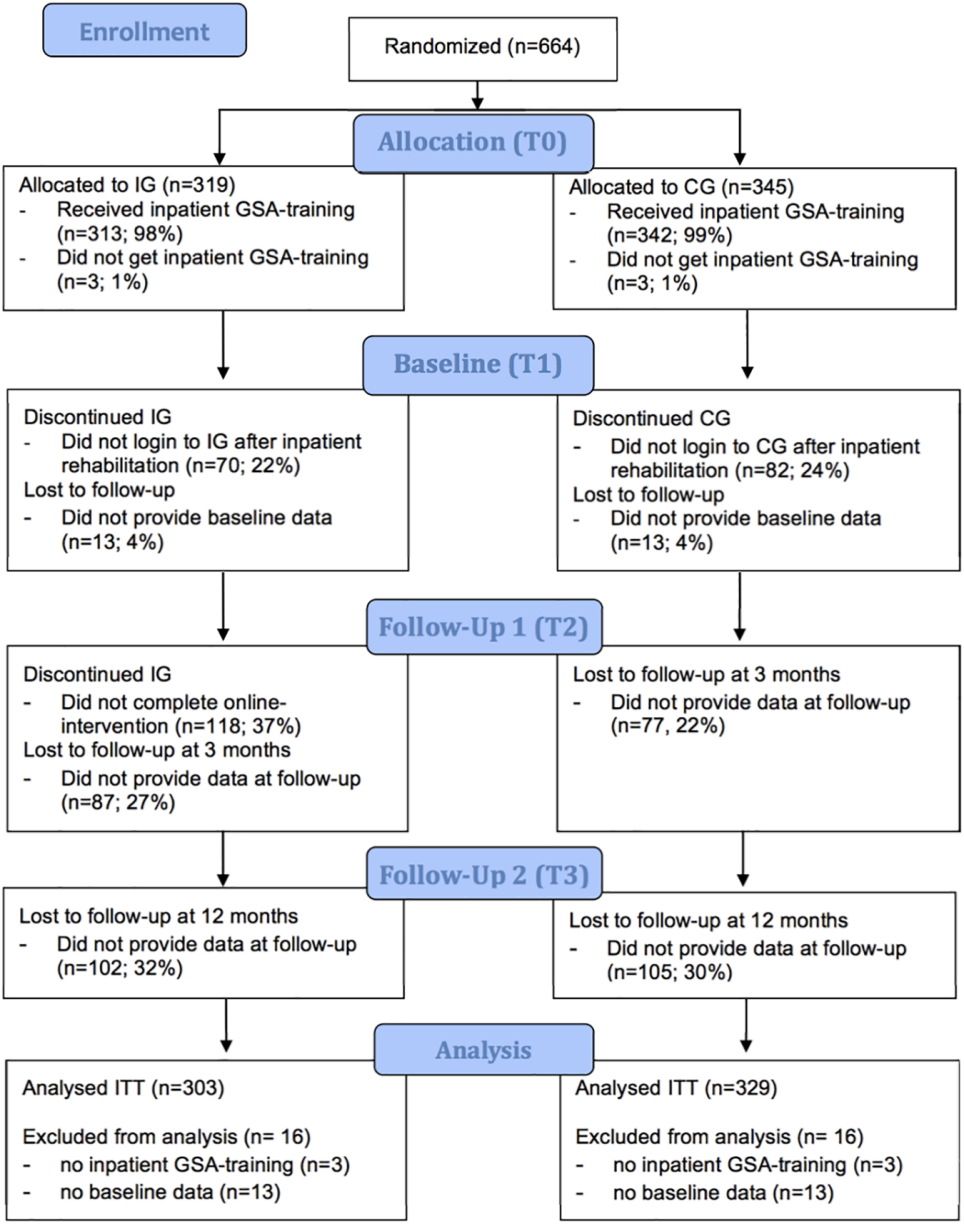
Participant flow after allocation and dropout throughout the study.

Patients were recruited during inpatient psychosomatic, orthopedic or cardiologic rehabilitation. As determined by brief screening upon intake into rehabilitation by the clinical staff, they were eligible if they a) were employed, b) reported high occupational stress, c) had a risk of early retirement or if they d) stated a high subjective need for occupational treatment. Furthermore they had to be e) German speaking, f) between 18 and 59 years old and g) they had to have private internet access. Comorbid mental disorders were not an inclusion criterion. When patients were eligible they received detailed verbal and written information about the study by the clinical staff. Because of organizational reasons in the participating clinics the number of patients not eligible could not be documented, therefore we could not include these data in the Consort flow chart (see Fig 1). After they had given their written informed consent patients willing to participate were assembled into groups of up to 12 participants, which were then randomized into one of the two conditions as described above.

Fig 1 shows the participant flow after allocation to the two groups and dropouts throughout the study.

A total of 664 patients were recruited in the four rehabilitation clinics. After obtaining written consent and screening, eligible patients were assembled into groups of up to 12 (minimum of two) participants. By means of cluster randomization we aimed to minimize confounding of the interventions by the interaction of patients during inpatient rehabilitation who were getting different instructions for the aftercare in the two randomized arms.

We excluded a total of 16 patients from the intervention as well as from the control group, as they did not receive the allocated intervention during inpatient rehabilitation, or did not provide baseline data. The intent-to-treat analyses included 303 cases in the intervention (IG) and 329 in the control group (CG). At the end of inpatient rehabilitation (baseline, T1) n = 70 (22%) participants were ‘non-starters’ regarding the online intervention, i.e. they didn’t login to the online platform after leaving the rehabilitation clinic, compared to n = 82 (24%) in the control group. During the three months following inpatient medical rehabilitation n = 118 (37%) participants of the IG discontinued and n = 128 (41%) completed the intervention, i.e. wrote a minimum of 6 blogs (post-hoc classification by median split).

Table 1 gives an overview of study participants at baseline (T1), separately for IG and CG.

**Table 1:** Sample description at baseline.

	IG (*n* = 303)				CG (*n* = 329)				Total (*N* = 632)				Sig.^a^^)^
	N	%	M	SD	N	%	M	SD	N	%	M	SD	
**Age**			48.5	7.13			48.9	7.2			48.7	7.2	1)
**Sex (male)**	164	54.1			179	54.4			343	54.3			2)
**Medical group**													3)
Cardiology	153	50.5			168	51.1			321	50.8			
Orthopedics	61	20.1			55	16.7			116	18.4			
Psychosomatics	89	29.4			106	32.2			195	30.9			
**Education**^**b**^^**)**^													4)
Secondary school (9 years)	39	13.0			60	18.4			99	15.8			
Secondary school (10 years)	122	40.7			125	38.3			247	39.5			
Grammar /High school (13 years)	139	46.3			141	43.3			280	44.7			
**Treatment duration (weeks)**^**c**^^**)**^			4.17	1.17			4.25	1.07			4.21	1.12	5)
**Work disability in the last 12 months (weeks)**			12.0	10.2			13.7	12.7			12.9	11.6	6)
**Work disability at discharge (‘yes’)**^**d**^^**)**^	105	35.7			137	41.9			242	39.0			7)
**Currently limited to not at all capable (‘yes’)**^**e**^^**)**^	240	82.8			274	86.4			514	84.7			8)
**Work ability permanently at risk (‘yes’)**^**f**^^**)**^	82	28.1			91	29.0			173	28.5			9)
**Plan to work till retirement age (‘yes’)**^**g**^^**)**^	168	58.3			169	53.5			337	55.8			10)
**Application for a pension filed/ planned (‘yes’)**^**h**^^**)**^	33	11.4			47	14.8			80	13.2			11)

IG = intervention group, CG = control group, M = mean, SD = standard deviation.

^a)^ χ^2^-tests resp. t-tests for independent samples

^b)^ n = missing in IG; n = 3 missing in CG

^c)^ n = 9 missing in IG; n = 2 missing in CG

^d)^ physician’s rating at discharge; n = 10 missing in IG; n = 3 missing in CG; ^e)-h)^ patient’s rating at discharge

^e)^ n = 13 missing in IG; n = 12 missing in CG

^f)^ n = 11 missing in IG; n = 15 missing in CG

^g)^ n = 15 missing in IG; n = 13 missing in CG

^h)^ n = 14 missing in IG; n = 11 missing in CG; 1) *t*_[630]_ = 0.61, *p* = 0.54; 2) χ_[1]_ = 0.01, *p* = .94; 3) χ_[2]_ = 1.43, *p* = .49; 4) χ_[2]_ = 3.43, p = .18; 5) *t*_[619]_ = 0.90, *p* = .39; 6) *t*_[630]_ = 1.87, *p* = .06; 7) χ_[2]_ = 2.48, *p* = .12; 8) χ_[1]_ = 1.58, *p* = .21; 9) χ_[1]_ = 0.06, *p* = .81; 10) χ_[1]_ = 1.44, *p* = .23; 11) χ_[1]_ = 1.50, *p* = .22.

The mean age of participants (ITT sample) was 49 years (25 to 59 years). There were slightly more males (54%) than females. About half of the patients came from cardiologic, about one third from psychosomatic and 18% from orthopedic rehabilitation. Psychosomatic patients were somewhat younger and had a lower level of education than the two somatic medical groups. The proportion of men was about two third in the cardiologic, one third in the orthopedic and under half in the psychosomatic group. Educational status was mostly medium or high in both groups. Treatment duration was about 4 weeks overall, with the longest duration in psychosomatic inpatient rehabilitation (Mean [M] = 5.33, Standard Deviation [SD] = 0.81) compared to the other two medical groups (cardiology M = 3.89, SD = 0.87; orthopedic M = 3.26, SD = 0.55; F_[2,618]_ = 283.67; p < .001).

All participants were employed in a part-time or full-time job. Nearly forty percent were rated unable to work at discharge by their physician, with an average duration of sick leaves for about 13 weeks in the last 12 months. A total of 85% considered their work capacity at least somewhat impaired, one third saw it as permanently endangered. While 56% of the participants expected to work until retirement, 13% had already applied for pension or planned to do so.

There was no difference between IG and CG in all these baseline variables, except for a trend to longer duration of ongoing work disability in the control group. In psychosomatic rehabilitation, work-disability in the past 12 months had lasted significantly longer than in the other medical groups (psychosomatic M = 20.03, SD = 15.50; cardiology M = 9.79, SD = 7.34; orthopedic M = 9.43, SD = 7.38; F_[2,629]_ = 64.69; p < .001).

### Treatments

#### Inpatient treatment

Inpatient rehabilitation was performed as usual. Under the guidance of a physician each group obtained multimodal treatment including assessment of functioning and medication, patient education, physical training, stress reduction, relaxation and psychological individual counseling as needed.

Additionally, over a period of two to three weeks, all participants were trained in stress management at the work place by four weekly interactive psycho-educative modules (90 minutes each). To standardize the procedure, the staff had been trained using a comprehensive manual in a detailed one-day seminar before study start, with a refresher half-way through recruitment. The first three sessions were identical for intervention and control group; participants and trainers remained blind to the result of randomization. Following unblinding, the trainer used the fourth module to prepare participants for their respective condition assigned. Each participant received a sealed envelope including the personal access data and a written description of the intervention or control program. After login, all patients were asked to change their password, to choose a nickname and to enter their e-mail address if they wanted to be informed by automated e-mails about study-related information, therapeutic tasks and questionnaires to be filled out.

#### Online intervention IG

The program itself adapted the model of supportive expressive therapy [27] to chronically ill identifying and working through maladaptive interpersonal cycles especially with colleagues or supervisors at the workplace. As a pure online intervention without personal contact our intervention does not qualify for psychotherapy under German legislation, therefore the intervention could be characterized as psycho-educative with a solid therapeutic background. Participants have been familiarized with the three components of Luborsky’s ‘Core Conflictual Relationship Theme (CCRT)’ and its recurrent and potentially maladaptive nature already during inpatient rehabilitation. On the internet-platform, participants were instructed to write down in detail interpersonal interactions in their current life with a focus on the workplace by means of weekly blogs. Writing down emotionally meaningful experiences was previously shown to exert positive effects on physical and mental health [28], e.g. coping with job loss could be improved [29]. Furthermore, writing is often used and widely accepted in online interventions [30]. Participants were instructed to select a certain day for blog writing and to spend 45 minutes every week to write blogs according to the instructions of the online therapist. Blogs were only shared between participants and their therapist, not with other participants. They got e-mail reminders on their ‘blog-day’ to login on the platform and to write a blog. With their consent, all blog contacts between therapist and patient were fully documented in the database, allowing subsequent content analyses. We provided individual feedback by the online therapist within 24 hours, therapists communicated with participants via the internet-platform. They gave standardized instructions to the participants in order to identify recurrent patterns of interpersonal experience at the workplace. Written interventions followed the model of CCRT [27] asking the participant to describe social encounters when returning from rehabilitation to daily work and social life according to his or her wish (W), the reactions of the others (RO) and reactions of the self (RS). In addition to providing general support, the online therapists drew the patients’ attention to potentially maladaptive patterns, which they had identified.

The two online therapists, trainees in psychodynamic psychotherapy, spent about 20 minutes per week per patient and they were not identifiable to the participants. Supervision was provided by a senior psychotherapist experienced in supportive expressive psychotherapy.

Further online features for the IG were a self-test with computer-generated feedback to the participants individual regarding ‘Pattern of Work-related Coping Behavior’ (short-form 44; AVEM-44) [31] and its change over time, audio samples with progressive muscle relaxation exercises, worksheets with information about stress-management techniques from the inpatient training group as well as a moderated patient forum, providing participants the opportunity to get in contact with former inpatients from their respective rehabilitation center. All these features were accessible for 12 weeks.

#### Online intervention CG

The control group received regular e-mail reminders to use selected information posted online about stress management and coping (physical activity, relaxation, healthy diet and sleep hygiene) over the same period of time. All information was permanently downloadable for participants. Overall there were six e-mail reminders, one every fortnight. One universal reminder was followed by specific reminders for the five different topics.

### Outcome measures

Depending on the technical equipment in the rehabilitation centers, T1 assessment was conducted online or paper-pencil in both conditions (intervention and control group). All other data were collected internet-based, unless the patients did not participate within two weeks. In this case, the patients got paper-pencil questionnaires by mail. The online assessment took about 45 minutes, comparable to the paper-pencil assessment. Patients got computer-generated feedback following each completed online questionnaire, describing the participants’ individual work-related behavior and experience pattern and its change over time, measured by the AVEM-44 [31]. The feedback was intended to serve as an incentive for participating in the survey.

The ‘Short Screening Instrument for the Assessment of Need for Occupation Related Treatment in Medical Rehabilitation’ (SIBAR) [32] consists of 15 items assessing objective indicators (current employment and periods of work disability and unemployment) and subjective indicators (reduced work ability, wish for pension). Items are answered by dichotomous (yes/no) and three to seven-point Likert-scales. Based on eight items, a risk index is calculated (ranging from 0 to 19) points; high scores were predictive of premature pension [32]. As it turned out, the SIBAR which was originally planned as primary outcome measure was not suitable, as items referring to work disability over the past 12 months did not fit our study design. Therefore we used the ‘**S**ubjective **P**rognosis of Gainful **E**mployment Scale’ (SPE) [33] as the primary outcome. In the original SPE-Scale, subjective prognosis of employment is assessed by three items: (1) ‘When you consider your current health and work ability: Do you believe that you will be working until retirement age?’ (Likert-scale 1 ‘sure’ to 5 ‘in no case’). (2) ‘Do you consider your work ability permanently threatened by your health status?’ (dichotomous: 1 ‘yes’ and 0 ‘no’) and (3) ‘Do you consider an application for premature pension?’ (dichotomous: 1 ‘yes’ and 0 ‘no’). After dichotomization of the first item all three items are added up resulting in a score between 0 and 3, so that a higher score indicates a higher risk for work disability or early retirement. The SPE only differed regarding the answering format of item 1 from the SIBAR which was already dichotomous (1 ‘yes’ and 0 ‘no’). A good internal consistency (Guttman scale coefficient of reproducibility = .99) and references for the validity of the scale (e.g. correlation with other measures of work performance or predictive validity concerning premature pension within the next five years) are documented [33].

Depressive symptoms were measured with the ‘Patient Health Questionnaire’ (PHQ-9), which quantifies the frequency of being bothered by each of the 9 diagnostic criteria of Major Depression over the past 2 weeks from 0 (‘not at all’) to 3 (‘nearly every day’). Responses are totalled to a score between 0 and 27 points. A PHQ-9 sum score of ≥ 10 is an indicator for the presence of any depressive disorder, with a sensitivity of 81% and a specificity of 82% [34, 35].

The ‘Generalized Anxiety Disorder Questionnaire’ (GAD-7) [36] was used to assess symptom severity of generalized anxiety. The GAD-7 is based on the most prominent diagnostic features of the DSM-IV diagnostic criteria for generalized anxiety disorder. Seven items are scored on a four-point Likert-scale as in the PHQ-9 with a total score ranging from 0 to 21. Reliability, validity and standard values were established by a representative German population sample (N = 5036; age 48 ± 18 years, 54% female, internal consistency of the GAD-7 was r = .89 [37].

Somatic symptoms severity was assessed with the 15 items of the PHQ-15 [38]. Items are scored on a three-point scale (not bothered = 0, bothered a little = 1, bothered a lot = 2) and the sum ranges between 0–30. Scores above 15 indicate high levels of somatic symptoms, respectively somatization severity.

Psychosocial stressors were assessed with the stress module (PHQ-10) of the PHQ-D [35], asking for being bothered in different aspects of daily life (e.g. financial situation, partnership, work, health status). The 10 Likert scaled items (not bothered = 0, bothered a little = 1, bothered a lot = 2) are totalled to a score ranging between 0 and 20 with a higher score indicating higher psychosocial distress [35].

Functional health status was assessed with the German version of the ‘Short Form Health questionnaire’ (SF-12) [39]. The SF-12 measures general health from the patients’ point of view with 12 items; scores were summed to a ‘Physical Component Summary’ scale (PCS), respectively a ‘Mental Component Summary’ scale (MCS) ranging from 0 to 100 (mean score of 50, standard deviation of 10) with higher scores indicating better physical or mental functioning.

The AVEM-44 [31] was used to assess patterns of work-related coping styles in three domains relevant to professional demands and health: work commitment, resistance to stress and emotions, i.e., subjective well-being. These domains are measured with 11 subscales, with each subscale consisting of four items. Items are rated on a Likert scale from 1 (strongly disagree) to 5 (strongly agree), with a maximum score of 30 points per subscale. Higher scores indicate higher levels of the scale-characteristic. The questionnaire has an acceptable to good reliability, with Cronbach’s α for the AVEM subscales ranging from 0.79 to 0.87. The validity of the AVEM has been examined in different groups, supporting its factorial structure [40, 41].

### Data analysis

All analyses have been conducted with IBM SPSS Statistics version 23 [42]. In order to test for differences at baseline, independent t-tests and χ^2^-tests have been conducted. Analyses on participation and dropout were done with χ^2^-tests and one ANOVA with medical group as independent and number of blogs as dependent variable. For the primary (SPE) and secondary outcomes, analysis of covariance were used to compare scores 12 weeks (T2) and 12 months after discharge of inpatient rehabilitation (T3) between the internet-based aftercare program (IG) and the augmented treatment as usual condition (CG), with covariates for medical group (psychosomatic, cardiologic, orthopedic) and baseline scores of the respective outcomes (T1). Between group effect sizes were calculated (Cohen’s d). Intention-to-treat analyses, as well as completer analyses were conducted. Missing data in the outcomes for the statistical analyses were imputed with Multiple Imputation (MI) using a Markov Chain Monte Carlo multivariate imputation algorithm with ten imputations. The proportions of missing data were about 2% at T0, 3.5% at T1, 27.7% at T2 and 31.6% at T3. All analyses were conducted at a two-sided level of significance of α = 0.05.

## Results

### Participation and dropouts in the different medical groups

In the IG 77% logged in to the website (CG 74%) and 65% of the IG wrote blogs. Patients from cardiologic rehabilitation logged in least frequently (67.9%) and orthopedic patients most frequently (87.9%); the psychosomatic group was in between (79.5%; Chi^2^_[__2__]_ = 21.12; p < .001). After logging in, at least one blog was written by 92.2% (orthopedic) vs. 89.9% (psychosomatic) vs. 76.0% (cardiology; Chi^2^_[__2__]_ = 8.75; p = .013).

The range of written blogs was 1 to 13, the mode was 9 blogs and the mean number of written blogs was 6 (SD = 4.21). The majority wrote blogs on more than half the occasions (>6 blogs). Number of blogs among those writing in the three medical groups varied statistically significantly between psychosomatic (M = 6.48; SD = 4.01) resp. orthopedic (M = 6.84; SD = 4.06) and cardiologic patients (M = 5.07; SD = 4.30; F_[__2__,229]_ = 4.09; p = .018). Other interventions offered were used less frequently. Almost 60% used the work sheets, about half of the patients used relaxation, only 20% contributed to the forum.

In the CG, about two thirds used the materials provided, the majority (56.5%) found them at least somewhat helpful, and downloaded materials (40–60% depending on the different topics) and reported of using them in the follow-up period (T3).

At the end of aftercare we asked both groups how helpful the intervention resp. the control condition was overall on a five-point Likert scale. In the dichotomized item 46% in the IG and 24% in the CG rated the intervention ‘quite’ or ‘very helpful’ and 54% in the IG resp. 76% in the CG rated the intervention ‘not at all’, ‘barely’ or ‘somewhat’ helpful (Chi^2^_[__1__, 420]_ = 22.76; p < .001).

### Primary and secondary outcomes

There were no differences regarding the primary outcome (SPE) between the two groups at baseline (IG: M = 0.81, SD = 0.99; CG: M = 0.91, SD = 1.00; n.s.). Regarding the different medical groups, SPE was higher in psychosomatic than in cardiologic rehabilitation (psychosomatic M = 0.99, SD = 1.07; cardiologic M = 0.77, SD = 0.96; orthopedic M = 0.89, SD = 0.95; F_[__2__,607]_ = 3.29; p = .039).

**Fig 2:**
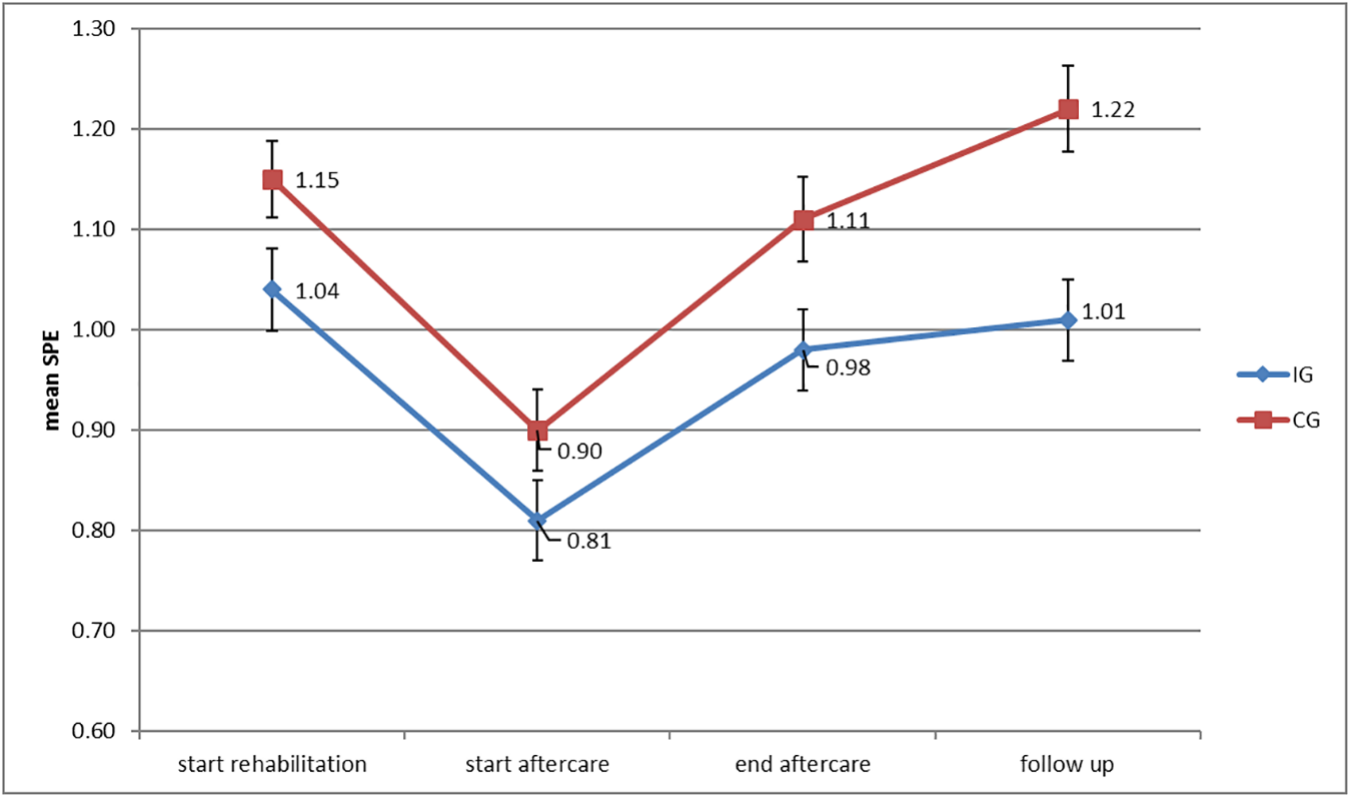
Primary outcome subjective prognosis of work ability over the course of time. SPE scale with a range of 0–3 (a higher score indicates a higher risk for work disability or early retirement); ANCOVA with SPE score at baseline (start aftercare) and indication as covariate at the end of aftercare (1) and at follow-up (2); (1) F_[1,606]_ = 2.38, p = .12, d = 0.11; (2) F_[1,606]_ = 4.8, p = .03, d = 0.18.

Fig 2 shows the primary outcome (SPE) in the IG and CG over the course of rehabilitation and aftercare. As Fig 2 shows, the subjective prognosis of employment was slightly more positive in the IG than in the CG at termination of aftercare and statistically significantly more positive at follow up. Between-group effect sizes, however, were low (d = .13 at termination and d = .20 at follow-up). Within-group effect sizes for the IG were d = 0.17 (T2) resp. d = 0.20 (T3) and for the CG d = 0.20 (T2) resp. d = 0.31 (T3). There was no significant effect of the covariate for the different medical groups.

The secondary outcomes depressive symptoms (PHQ-9), anxiety (GAD-7), somatization (PHQ-15), psychosocial stressors (PHQ-10) and functional health status (SF-12) were comparable in both groups at baseline (see Table 2) but there were several differences between the three medical groups, mainly with higher scores for the psychological complaints within the psychosomatic inpatients.

**Table 2:** Means (M) and standard deviations (SD) of distress outcome variables at baseline (T1), post-treatment (T2) and six month follow-up (T3) and differences between groups at the end of treatment (T2) and at six month follow-up (T3).

Outcome	T1				T2							T3						
	IG (N = 303)		CG (N = 329)		IG (N = 303)		CG (N = 329)					IG (N = 303)		CG (N = 329)				
	M	SD	M	SD	M	SD	M	SD	F	p	d	M	SD	M	SD	F	p	d
**PHQ-9**	8.56	5.94	8.40	5.52	7.96	5.34	8.93	5.61	8.34	**.017**	**0.18**	7.86	5.31	8.84	5.73	8.12	**.005**	**0.18**
**GAD-7**	6.75	5.12	6.29	4.97	6.69	4.62	7.25	4.93	6.40	**.026**	**0.12**	6.35	4.46	7.51	4.80	16.77	**< .001**	**0.25**
**PHQ-15**	10.58	5.57	10.32	5.31	9.77	5.29	9.99	5.44	1.47	.320	0.04	9.88	5.46	10.30	5.53	3.16	.126	0.08
**PHQ-10**	7.80	4.41	7.42	4.21	7.19	4.17	7.55	4.14	4.53	**.050**	**0.09**	7.13	3.96	7.74	4.35	9.08	**.006**	**0.15**
**SF-12 psych.**	39.83	11.78	39.39	11.46	42.91	11.15	40.61	11.74	6.66	**.023**	**0.20**	43.64	11.68	41.26	11.62	6.63	**.026**	**0.20**
**SF-12 phys.**	42.75	9.65	42.72	9.95	43.77	9.96	44.14	10.22	0.71	.558	0.04	44.10	10.26	44.20	10.20	0.65	.485	0.01

PHQ-9: depressive symptoms, GAD-7: anxiety, PHQ-15: somatoform symptoms, PHQ-10: psychosocial stressors, SF-12 psych.: mental health status, SF12 phys.: physical health status; T1 = Baseline; T2 = End of aftercare; T3 = follow up; IG = Intervention Group; CG = Control group; M = mean, SD = standard deviation; F = F-statistics of the ANCOVA with scale-score at baseline and medical group as covariates; d = Between-group effect size Cohen’s d.

Table 2 also displays the secondary outcomes concerning distress at the end of aftercare (T2) and at follow up (T3). Depressive symptoms (PHQ-9), anxiety (GAD-7) and psychosocial stressors (PHQ-10) decrease further during and after online aftercare in the IG, whereas they remained stable or went back up slightly in the CG. Mental health status (SF-12 psych) increased in the IG at the end of aftercare as well as in the follow-up and increased in the CG. Between-group effect sizes for the statistically significant differences varied between d = .09 (PHQ-10 T2) and d = .25 (GAD-7 T3). We observed statistical significant effects for the covariate medical groups for some of these secondary outcomes.

Patterns of work-related coping styles, assessed with the AVEM-44 were comparable in the IG and CG at baseline. In Table 3 the scores over the course of rehabilitation and after care are documented for both groups. Statistical significant differences in favour of the IG were found at the end of aftercare for the scales ‘Experience of success at work (EW)’, ‘Inner calm and balance (IC)’ and ‘Satisfaction with life (SL)’. For ‘Inner calm and balance (IC)’ this was also the case in the follow-up (Table 3). Between-group effect sizes varied between d = .20 (AVEM-EW T2) and d = .25 (AVEM-IC T2).

**Table 3:** Means (M) and standard deviations (SD) of work-related (AVEM) outcome-criteria at baseline (T1), post-treatment (T2) and six month follow-up (T3) and differences between groups at the end of treatment (T2) and at six month follow-up (T3).

Outcome AVEM	T1				T2							T3						
	IG (N = 303)		CG (N = 329)		IG (N = 303)		CG (N = 329)					IG (N = 303)		CG (N = 329)				
	M	SD	M	SD	M	SD	M	SD	F	p	d	M	SD	M	SD	F	p	d
**SI**	4.70	2.05	4.73	2.13	4.46	1.81	4.46	1.94	0.25	.697	0.01	4.43	1.75	4.39	1.84	0.57	.507	0.02
**WA**	4.51	1.99	4.35	1.99	4.31	1.77	4.18	1.87	0.26	.673	0.07	4.12	1.78	4.14	1.82	1.35	.365	0.01
**WE**	5.75	2.14	5.87	2.16	5.20	1.98	5.24	2.07	0.29	.638	0.02	4.92	2.03	5.12	2.12	1.53	.380	0.10
**SP**	5.19	2.11	5.30	2.10	4.81	1.90	4.97	1.95	0.75	.464	0.08	4.72	1.94	4.91	1.93	1.28	.427	0.10
**DA**	5.06	1.24	5.16	1.26	5.06	1.23	4.89	1.23	3.98	.142	0.14	4.93	1.26	5.06	1.22	1.91	.277	0.11
**TR**	5.44	1.96	5.63	1.99	5.06	1.93	5.30	1.65	1.19	.340	0.13	5.03	1.93	5.21	1.98	0.37	.603	0.09
**PP**	3.80	1.86	3.85	2.03	3.75	1.73	3.67	1.84	0.22	.429	0.05	3.58	1.79	3.64	1.73	0.46	.589	0.03
**IC**	4.08	1.72	3.97	1.68	4.12	1.65	3.73	1.52	10.39	**.004**	**0.25**	4.17	1.69	3.82	1.55	7.40	**.041**	**0.22**
**EW**	4.13	2.10	4.05	2.10	4.25	1.95	3.85	1.99	10.98	**.003**	**0.20**	4.18	1.89	3.96	1.97	2.47	.164	0.11
**SL**	3.62	1.75	3.35	1.78	3.65	1.66	3.26	1.73	4.78	**.036**	**0.23**	3.62	1.68	3.33	1.65	1.99	.303	0.17
**ES**	3.69	1.40	3.70	1.42	3.44	1.24	3.25	1.20	4.92	.080	0.16	3.31	1.26	3.32	1.24	0.53	.609	0.01

AVEM-SI: subjective importance of work; AVEM-WA: work-related ambition; AVEM-WE: willingness to work until exhausted; AVEM-SP: striving for perfection; AVEM-DA: distancing ability; AVEM-TR: tendency to resignation; AVEM-PP: proactive problem-solving AVEM-IC: inner calm and balance; AVEM-EW: experience of success at work; AVEM-SL: satisfaction with life; AVEM-ES: experience of social support;; T1 = Baseline; T2 = End of aftercare; T3 = follow up; IG = Intervention Group; CG = Control group; M = mean, SD = standard deviation; F = F-statistics of the ANCOVA with scale-score at baseline and medical group as covariates; d = Between-group effect size Cohen’s d.

## Discussion

Regardless of the specific diagnosis, chronic disease is often fraught with comorbid common mental disorders such as anxiety or depression increasing difficulties of returning to work after prolonged sickness absences [43]. Rehabilitation may then be indicated in order to restore physical and mental work capacity [43], however, even after successful rehabilitation, return to work is often difficult, when extended sickness absences have eroded patients’ sense of competence and led to fears of failure and rejection by colleagues. Unlike usual ‘offline’ aftercare by individual or group interventions, online aftercare may be more easy to integrate into daily routines, as participants are free to choose convenient times and save transportation time and costs. We therefore devised and evaluated a novel transdiagnostic psychodynamic online aftercare following psychosomatic, cardiological, or orthopedic inpatient rehabilitation. In a randomized controlled trial we aimed to enhance subjective prognosis of gainful employment and quality of life and reduce anxiety and depressive symptoms in the critical period of return-to-work.

The weekly writing intervention adapted a supportive expressive therapy approach. Participants were instructed to write down their interpersonal experiences returning to work according to their wish, reactions of the others and reactions of the self. Therapist feedback aimed at supporting participants and improving work-related attitudes by identifying and addressing maladaptive interpersonal interactions as they applied to workplace interactions. The control group received regular reminders of online materials regarding stress and health-related issues.

Overall, acceptance of the trial and of the psycho-educative online interventions was good. Demographic characteristics (e.g. higher age in the predominantly male cardiologic group), as well as the scope and duration of inpatient rehabilitation differed in the diagnostic subgroups. While overall acceptance was good, cardiologic patients used the intervention least frequently and orthopedic patients most frequently. We assume that the fact that patients of cardiologic rehabilitation could not log in during inpatient treatment may have reduced their participation in the study, and their intensity of using the intervention considering the lower number of written blogs compared to psychosomatic and orthopedic patients. Regarding the pattern of use, the majority of participants (59%) wrote more than 6 blogs, and almost half (44%) wrote 9 and more blogs.

As we had hypothesized, compared to the control group, the intervention group improved significantly regarding their subjective prognosis for gainful employment after inpatient rehabilitation, but only reached the level of significance at the 6-month follow-up, i.e. 12 months after inpatient rehabilitation. Between-group effect sizes were low and inspection of within-group effect sizes showed that patients of the CG deteriorated more concerning their subjective prognosis of gainful employment than patients of the IG. There were also significant improvements regarding depressive symptoms, anxiety, stress, psychological quality of life and quality of life as well as experience of success at work resp. inner calm subscales from the AVEM, already evident at treatment termination. Thus, reduction of distress may precede restoration of subjective work capacity. Since we included a sample with work-related problems that included persons with long sick leaves and enduring impairment, it would have been interesting to assess work-related anxieties, often associated with longer sick leaves duration, premature pension or job loss [44, 45].

The fact that our findings for the primary outcome were significant taking into consideration baseline scores and the respective medical groups as covariates shows that the treatment was effective across different mental and physical diseases. This is encouraging given that the medical groups also differed regarding demographic characteristics (e.g. sex, education, duration of sick leave), duration and scope of medical rehabilitation.

### Limitations

The number of patients who were not eligible for the trial could not be documented because of organizational reasons in the participating clinics. Therefore we could not describe in detail, how the study sample differs from the regular sample of patients treated in the participating clinics. But from descriptive inspection of the basic demographic and clinical characteristics we could say that our sample is representative for the relevant clinical settings (e.g. age, sex ratio or treatment duration) of inpatient medical rehabilitation in Germany, as could be seen in the quality reports of the German Statutory Pension Insurance Scheme [46].

In the process of the trial, it turned out that out of the range of interventions we had offered (i.e. blog, work sheets, forum), the writing task was used most. While blogs were used most and we found some evidence of an association between blog writing and outcome, we cannot preclude that other, less- used interventions may have had an additional effect.

Overall, while our findings were robust, they were also small in effect sizes. This may be due to three factors: (a) The findings must be evaluated against the background of about four weeks of intensive inpatient rehabilitation and an empirically tested group training of coping with work-related stress received both by the intervention and the control group. Furthermore depressive symptoms and anxiety scores at the beginning had decreased significantly at the end of rehabilitation, when our intervention started. (b) Using treatment as usual condition, the previous trial of Ebert et al. [17] found that the control group deteriorated significantly, while the intervention group remained stable or improved during the follow-up period. Our control group received an additional written intervention with detailed biweekly information pertaining to stress and health, so we can speak of an augmented TAU condition. These materials were read and downloaded by a considerable proportion of participants. This may help to explain, why the participants in our control group showed no evidence of significant deterioration as in the previous trial, thus reducing the comparative effect sizes of the intervention. (c) Introducing the online interventions by group sessions makes its use cumbersome for the rehabilitation clinic. Despite the training we conducted in the clinics, we cannot be sure whether these groups were conducted in the same way, and whether participants were prepared and motivated not only to log in, but also to follow through with the intervention. The differential log in rates may indicate that patients of cardiologic rehabilitation may need a better rationale and motivation.

## Conclusions and perspective

Overall psychodynamic online aftercare was well accepted and outperformed the augmented TAU condition across a variety of chronic mental and somatic conditions as expected, albeit with small effect sizes. In our current research we assess differential applicability and generalizability. We are currently embarking on a new trial transferring the online interventions into regular aftercare including an even wider range of diseases (plus cancer). Further analyses will explore differential findings between the three medical groups in depth, as the covariate medical group was significant in some analyses.

Based on the experiences and the verbatim accounts of participants, we are currently creating an online platform with videos of actors recounting their online aftercare experiences and experts explaining the rationale and the benefit of the online intervention. In order to increase effectiveness, sustained participation is strongly advocated. Referral to the program resides in the hands of the physicians who also prescribe other kinds of aftercare.

## Supporting information

S1 File SPSS-Data. (SAV)

S2 File CONSORT checklist. (PDF)

S3 File Study protocol submitted to the local ethics committee. (PDF)
