# Supplementary material for: Evaluation of a transdiagnostic psychodynamic online intervention to support return to work: A randomized controlled trial
Source: PLoS One. 2017 May 8;12(5):e0176513. doi: 10.1371/journal.pone.0176513 (PMC5421767; doi:10.1371/journal.pone.0176513)
Supplement: S3 File — (PDF) [file pone.0176513.s003.pdf]

An die
Landesärztekammer Rheinland-Pfalz
Ethikkommission
Deutschhausplatz 3
55116 Mainz

Antrag

an die Ethik-Kommission
der Landesärztekammer Rheinland-Pfalz
zur ethischen Begutachtung eines medizinischen Forschungsvorhabens
am Menschen

1 Allgemeine Angaben

1.1 Datum der Antragstellung

20.10.2010 (Erstantrag), 05.01.2011 (1. Überarbeitung)

1.2 Titel des Forschungsvorhabens

Entwicklung und Evaluation einer indikationsübergreifenden internetbasierten Nachsorgemaßnahme zur Verbesserung der beruflichen Reintegration

Förderkennzeichen: 0423/00-40-65-50-25

1.3 Verantwortliche Studienleiter und MitarbeiterInnen

Dipl.-Psych. Dr. Rüdiger Zwerenz
Klinik für Psychosomatische Medizin und Psychotherapie, Universitätsmedizin der Johannes Gutenberg-Universität Mainz, Untere Zahlbacher Str. 8, 55131 Mainz
e-mail: ruediger.zwerenz@unimedizin-mainz.de

Weitere Projektleiter: Prof. Dr. Manfred E. Beutel; Jens Edinger

Weitere MitarbeiterInnen: Dipl.-Psych. Katharina Gerzymisch; cand. Psych. Daniela Rudolph

Klinik für Psychosomatische Medizin und Psychotherapie, Universitätsmedizin der Johannes Gutenberg Universität Mainz, Untere Zahlbacher Str. 8, 55131 Mainz

1.4 Verantwortlicher Sponsor
Deutsche Rentenversicherung Bund (DRV-Bund), Geschäftsbereich Sozialmedizin und Rehabilitation, Ruhrstr. 2, 10709 Berlin (Ansprechpartnerin: Frau Katharina Wirtgen)

1.5 Es handelt sich um eine multizentrische Studie.

1.6 Sonstige Teilnehmer
- CA Dr. med. Martin Holme, DRV Reha-Zentrum Bad Pyrmont, Klinik Weser, Schulstr. 2, 31812 Bad Pyrmont
- CA Dr. med. R.J. Knickenberg, Psychosomatische Klinik Bad Neustadt der Rhön-Klinikum AG, Abteilung Rehabilitation, Salzburger Leite 1, 97616 Bad Neustadt/Saale
- CÄ Dr. med. Sieglinde Spörl-Dönch, Klinik „Haus Franken GmbH", Fachklinik für Prävention und Rehabilitation von Herz-, Kreislauf- und Gefäßerkrankungen, Salzburger Leite 1, 97616 Bad Neustadt/Saale
- CA Dr. med. U. Kiwus, DRV-Reha-Zentrum Bad Nauheim, Klinik Wetterau, Zandersstr. 30-32, 61231 Bad Nauheim

1.7 Orte der Durchführung des Forschungsvorhabens
- DRV Reha-Zentrum Bad Pyrmont, Klinik Weser, Schulstr. 2, 31812 Bad Pyrmont
- Psychosomatische Klinik Bad Neustadt der Rhön-Klinikum AG, Abteilung Rehabilitation, Salzburger Leite 1, 97616 Bad Neustadt/Saale
- Klinik „Haus Franken GmbH", Fachklinik für Prävention und Rehabilitation von Herz-, Kreislauf- und Gefäßerkrankungen, Salzburger Leite 1, 97616 Bad Neustadt/Saale
- DRV-Reha-Zentrum Bad Nauheim, Klinik Wetterau, Zandersstr. 30-32, 61231 Bad Nauheim

1.8 Finanzierung
Die Studie wird mit Mitteln der Deutschen Rentenversicherung Bund (DRV-Bund) finanziert.

1.9 Anzeige der Prüfung
Entfällt, da keine Arzneimittelprüfung.

1.10 Das Forschungsvorhaben wurde noch keiner anderen Ethik-Kommission zur Begutachtung vorgelegt.

2 Begründung des Forschungsvorhabens
2.1 Ziele des Forschungsvorhabens
Gegenstand des Projekts ist die Entwicklung und Evaluation einer indikationsübergreifenden internetbasierten Nachsorgemaßnahme zur Bearbeitung beruflicher Problemlagen in der medizinischen Rehabilitation. Es soll die Fragestellung untersucht werden, ob eine internetbasierte Nachsorgemaßnahme die berufliche Reintegration von beruflich belasteten Patienten der stationären kardiologischen, orthopädischen und psychosomatischen Rehabilitation dauerhaft verbessert.
Als Hypothese wird angenommen, dass durch die Teilnahme an einer internetbasierten Nachsorgemaßnahme die während der stationären medizinischen Rehabilitation erzielten Verbesserungen der Bewältigungsmöglichkeiten im Umgang mit beruflichen Belastungen stabilisiert werden und dadurch die berufliche Reintegration gefördert wird.

2.2 Gründe für die Durchführung, Problemdarstellung
Aktuell gibt es noch kaum Befunde dazu, wie der Transfer der während stationärer Maßnahmen der medizinischen Rehabilitation erreichten Veränderungen in den Berufsalltag möglich ist. In unserer Arbeitsgruppe wurden verschiedene indikationsübergreifende berufsbezogene Module für die stationäre medizinische Rehabilitation entwickelt und evaluiert. Allerdings war in keiner der bisher

durchgeführten Studien eine Vernetzung mit ambulanten Nachsorgemaßnahmen möglich. Dadurch kam es – wie wir in unseren katamnestischen Nacherhebungen zeigen konnten – bei der Wiedereingliederung vielfach zu Schwierigkeiten, z.B. durch veränderte Aufgabenbereiche, Verfahren und Technologien oder Konflikte bei der Reintegration ins Team nach längerer krankheitsbedingter Abwesenheit. An diesen kritischen Punkten sind die Rehabilitanden auf sich gestellt und es gelingt vielen nicht, die während der stationären Rehabilitation erlernten Strategien zielführend anzuwenden. Wenn die berufliche Reintegration dann misslingt, kommt es gehäuft zu erneuter Symptomverschlechterung, Krankschreibung und letztlich Rentenbegehren.

2.3 Stand der Wissenschaft

Arbeitspsychologisch ist gut belegt, dass belastende Arbeitsmerkmale einen direkten Einfluss auf subjektive Belastungswahrnehmungen, psychosomatische Beschwerden und somatische Erkrankungen haben (Frese, 1991; Siegrist, 2002). Dies spiegelt sich beispielsweise in Selbsteinschätzungen von Patienten in der stationären psychosomatischen Rehabilitation wider (Beutel, Zwerenz, Kayser, Schattenburg, & Knickenberg, 2004), die sich beruflich stärker belastet fühlen und über geringere Ressourcen verfügen als vergleichbare Personen aus der Allgemeinbevölkerung (Zwerenz, Knickenberg, Schattenburg, & Beutel, 2004). Lange Arbeitsunfähigkeitszeiten und subjektive Einschätzungen der beruflichen Leistungsfähigkeit zählen indikationsübergreifend zu den stärksten und konsistentesten negativen Vorhersagemerkmalen für den Erfolg von Maßnahmen der medizinischen Rehabilitation (Beutel, et al., 1999; Irle, Klosterhuis, & Grünbeck, 1998; Winkelhake, Schutzeichel, Niemann, & Daalmann, 2003).
Die medizinische Rehabilitation soll die Teilhabe am sozialen und beruflichen Leben sichern. Auf den Bedarf an speziellen berufsbezogenen Behandlungsangeboten wurde wiederholt hingewiesen (BAR, 2002; Beutel, et al., 1999; BfA, 2000; U. Koch, Bürger, Schulz, Glier, & Rodewig, 1997; VDR, 1992). Aktualität gewinnen berufsbezogene Belastungen und Konflikte von chronisch Kranken durch den rapiden gesellschaftlichen und technologischen Wandel der Arbeitsanforderungen sowie durch Arbeitsplatzunsicherheit und Arbeitslosigkeit. Wie die aktuellen Statistiken der Krankenkassen (z.B. DAK, 2010) zeigen, kam es zu einer erheblichen Zunahme psychomentaler Belastungen von Arbeitnehmern in den letzten Jahren.
In aktuellen Konzepten der stationären Rehabilitation gewinnen berufsbezogene Behandlungsangebote zum Erhalt bzw. zur Wiederherstellung der Erwerbsfähigkeit einen zunehmenden Stellenwert (Müller-Fahrnow, Greitemann, Radoschewski, Gerwinn, & Hansmeier, 2005). Mittlerweile liegen diagnostische Instrumente zur Erfassung beruflicher Problemlagen sowie zur berufsbezogenen Motivation (Bürger & Deck, 2009; Fiedler, Ranft, Greitemann, & Heuft, 2005; Löffler, Wolf, & Vogel, 2008; Streibelt, 2009; Zwerenz, Knickenberg, Schattenburg, & Beutel, 2005) vor. Ebenso existieren bewährte Behandlungskonzepte wie die berufliche Belastungserprobung (Beutel, et al., 2005) sowie neue berufsbezogene Gruppeninterventionen für verschiedene Zielgruppen und Indikationsbereiche (Beutel, et al., 2002; Beutel, Gerhard, et al., 2004; Beutel, et al., 2006; S. Koch, Hedlund, Rosenthal, & Hillert, 2006; Rudolf & Heitzmann, 2006). Die Studien ergaben indikationsübergreifend eine gute Umsetzbarkeit und Akzeptanz der spezifischen Behandlungsmaßnahmen, die sich an beruflichen Belastungen der Patienten orientieren, Motivation fördern und berufliche Einstellungen verändern (Beutel, et al., 2002; Beutel, Gerhard, et al., 2004; Beutel, et al., 2006; Beutel, et al., 2005; Hillert, et al., 2007; Wagner, et al., 2008; Wagner, et al., 2006).
Internetbasierte Nachsorgemaßnahmen sind vielversprechend, da erste kontrollierte Studien die Wirksamkeit von internetbasierten Therapie- bzw. Beratungsangeboten für unterschiedliche psychische Störungen und Lebensprobleme belegen (Bee, et al., 2008). Darüber hinaus eröffnet eine internetbasierte Therapie besonders Menschen mit erschwertem Zugang zu einer herkömmlichen Behandlung neue Möglichkeiten (Kersting, Schlicht, & Kroker, 2009). Laufende Forschungsprojekte zur Evaluation internetgestützter Reha-Nachsorge weisen auf eine hohe

Patientenzufriedenheit und Akzeptanz des Internets als Nachsorgemedium hin (Golkaramnay, Cicholas, & Vogler, 2010; Theissing & Deck, 2009).

2.4 Eigenschaften der Prüfsubstanz

Entfällt

3 Allgemeine Planung

3.1 Zusammenfassende Darstellung des Studienprotokolls

In die Studie aufgenommen werden konsekutiv alle beruflich belasteten Patienten, die ihr schriftliches Einverständnis zur Studienteilnahme geben und im Screening zu Beginn der stationären Rehabilitationsmaßnahme eine berufliche Belastungssituation erkennen lassen.

Patienten, die mit dem Studienablauf einverstanden sind und die Einschlusskriterien erfüllen, werden per Randomisierung der Interventionsgruppe (IG) oder der Kontrollgruppe (KG) zugewiesen. Beide Gruppen erhalten Zugang zu einer durch das Studienzentrum der Universitätsmedizin Mainz verwalteten Internetplattform. Bei Anmeldung auf der Plattform werden die Patienten gebeten, eine E-Mail-Adresse oder Telefonnummer anzugeben, über die sie automatisch benachrichtigt werden, wenn Informationen, therapeutische Aufgaben oder auszufüllende Fragebögen für Sie hinterlegt sind.

Patienten der IG erhalten während der stationären Rehabilitation das „Gesundheitstraining Stressbewältigung am Arbeitsplatz – GSA“[1] und im Anschluss an die stationäre Rehabilitation das mit der stationären Behandlung vernetzte internetbasierte Nachsorgeangebot. Aufbauend auf das stationäre Modul erhält die IG drei Monate lang über die Plattform eine internetbasierte Nachsorgemaßnahme mit Arbeitsmaterialien, Entspannungsübungen und einer strukturierten Schreibaufgabe, im Sinne eines wöchentlichen Berichts, der durch den Online Therapeuten kommentiert wird. Zum Ende der Intervention (= drei Monate poststationär) sowie insgesamt 12 Monate nach Entlassung aus der stationären Rehabilitation wird die IG nachbefragt.

Patienten der KG erhalten ebenso das stationäre Angebot „GSA“ und werden über die bereits vorhandenen Möglichkeiten der ambulanten Reha-Nachsorge sowie Psychotherapie informiert („Treatment as usual“). Im Sinne eines Aufmerksamkeitsplacebos werden allen Teilnehmern der KG ab Ende der stationären Rehabilitation für die Dauer von drei Monaten regelmäßig über die Internetplattform Links zur Verfügung gestellt, die sie zu allgemein zugänglichen Informationen zur Nachsorge und Materialien zum Umgang mit beruflichen Belastungen führen. Ebenso werden sie poststationär drei und 12 Monate nachbefragt.

3.2 Geplanter Beginn und voraussichtliche Dauer des Forschungsvorhabens

Das Projekt ist entsprechend dem Bescheid der DRV-Bund vom 03.09.2010 für die Dauer von drei Jahren ab dem 01.11.2010 bis 31.10.2013 bewilligt. Davon sind 1 ½ Jahre für die Patientenrekrutierung mit Randomisierung und darauf folgender Intervention vorgesehen. Im weiteren Verlauf erfolgen die Nachbefragungen drei Monate nach Entlassung aus der stationären Rehabilitation (Ende der Nachsorgemaßnahme) sowie neun Monate später (Katamnese, 12 Monate nach Entlassung aus der Klinik), Auswertung und Publikation. Der Beginn der Patientenrekrutierung ist für April 2011 geplant. Die Entwicklung der Intervention und die Vorbereitung der Datenerhebung werden bis dahin abgeschlossen sein.

3.3 Untersuchung an gesunden Probanden

Entfällt

---

[1] http://www.zentrum-patientenschulung.de/datenbank/steckbrief.php?schulungen_id=113
http://forschung.deutsche-rentenversicherung.de/ForschPortalWeb/ressource?key=Manual_%20Gesundheitstraining_am_Arbeitsplatz_GSA.pdf

3.4 Untersuchungen an Patienten

Es sollen insgesamt $N = 800$ Patienten eingeschlossen werden, davon $n = 400$ Teilnehmer der IG ($n = 100$ je Kooperationsklinik) und $n = 400$ Teilnehmer der KG ($n = 100$ je Kooperationsklinik).

Vor Beginn der Patientenrekrutierung wird zentral durch das Studienzentrum in Mainz und durch die einzelnen Kooperationskliniken ein Votum von den entsprechenden Landesethikkommissionen und die Zustimmung des Datenschutzbeauftragten der DRV-Bund eingeholt. Im Rahmen des Projekts sollen über schriftliche und elektronische Patientenbefragungen Daten zu beruflichen Belastungen, zur Symptombelastung und Behandlungszufriedenheit erhoben werden, ebenso sollen die in den Kliniken durch die Behandler dokumentierten somatischen und psychischen Diagnosen sowie die Basis-Behandlungsdaten verwendet werden. Im Verlauf, d.h. nach der stationären Rehabilitationsmaßnahme, werden die teilnehmenden Patienten über die Internetplattform – auf Wunsch auch per Post – zu beruflichen Problemlagen und Symptombelastungen sowie zum Gelingen der beruflichen Reintegration befragt. Alle Patienten (Interventions- und Kontrollgruppe) werden mündlich wie schriftlich über die Durchführung der Befragungen (einschließlich der Katamnese), die Zuweisung zu den Studiengruppen (Randomisierung) sowie über die Versuchsbedingungen aufgeklärt. Patienteninformationen mit schriftlicher Einwilligung werden entsprechend dem Datenschutzkonzept der DRV-Bund den Patienten vorgelegt.

Als Messgrößen werden quantitative Daten bei Aufnahme, im Verlauf, bei Entlassung, zum Ende der Intervention (nach drei Monaten) sowie 12 Monate nach Entlassung mit standardisierten Verfahren zu soziodemographischen, berufs- und gesundheitsbezogenen Merkmalen erhoben (vgl. dazu Zielgrößen unter 3.1). Die Datenerhebung erfolgt sowohl in der IG als auch der KG durch eine elektronische Patientenbefragung. Auf Wunsch werden die Fragebögen auch in einer Papierversion vorgelegt und zur Katamnese per Post versandt. Sowohl in der IG als auch der KG wird das Inanspruchnahmeverhalten zu den geplanten Messzeitpunkten ausführlich dokumentiert, indem erhoben wird, welche anderen Nachsorgemaßnahmen (Maßnahmen zur Stressbewältigung, sowie zur Bewältigung beruflicher Belastungen, Arztbesuche, Psychotherapie, Medikamente, etc.) von den Patienten in Anspruch genommen wurden.

Der zeitliche Aufwand beträgt für die verschiedenen Messzeitpunkte jeweils 30-60 Minuten zur Bearbeitung der Fragebögen. Die stationäre Intervention (GSA-stationär) wird sowohl für Patienten der IG als auch der KG in die Rehabilitationsbehandlung integriert sein, so dass den Patienten kein zusätzlicher Zeitaufwand entsteht. Im Anschluss an die Behandlung werden die Teilnehmer der IG über drei Monate für ca. 60 Minuten/Woche (darüber hinaus ist der Zeitaufwand durch Nutzung der Informationsplattform für Patienten selbständig zu bestimmen) an der internetbasierten Nachsorgemaßnahme teilnehmen. Patienten der KG werden ebenfalls wöchentlich einen vergleichbaren Zeitaufwand von ca. 60 Minuten haben, sofern sie die Ihnen zur Verfügung gestellten Informationen nutzen.

3.5 Art der Prüfung

Es handelt sich um die Wirksamkeitsprüfung einer internetbasierten berufsbezogenen Nachsorgemaßnahme hinsichtlich der beruflichen Reintegration von Patienten mit beruflichen Problemlagen.

3.6 Studienart

Es handelt sich um eine prospektive kontrollierte randomisierte Interventionsstudie. Verglichen werden Patienten der stationären medizinischen Rehabilitation (Kardiologie, Orthopädie, Psychosomatik) mit beruflichen Belastungen, die randomisiert einer internetbasierten Nachsorgemaßnahme zugewiesen werden, und Patienten unter „treatment as usual“ Behandlung mit Aufmerksamkeitsplacebo (vgl. 3.1).

Da nicht in allen Kliniken sichergestellt werden kann, dass durchgängig zwei Gruppen (IG und KG) parallel mit dem stationären Interventionsprogramm geschult werden können, erfolgt eine Cluster-

Randomisierung, d.h. Patienten werden entsprechend der Ein- und Ausschlusskriterien in Gruppen aufgenommen, die anschließend per Zufall der Interventions- oder der Kontrollbedingung zugeordnet werden. Die Randomisierung erfolgt extern durch eine unabhängige interdisziplinäre Einrichtung, das „Studienzentrum Psychische Erkrankungen“ (BMBF gefördert), einer Untereinheit des „Interdisziplinären Zentrums Klinische Studien“ in Mainz.

3.7 Arzneimittelprüfung
Es handelt sich nicht um eine Arzneimittelprüfung.

3.8 Monitoring und Audit
Die Supervision und Qualitätskontrolle der in den Kooperationskliniken durchgeführten stationären Schulungsmaßnahmen sowie der in der Nachsorge durchgeführten internetbasierten Nachsorgemaßnahmen erfolgt durch die Projektleiter im Studienzentrum.

3.9 Prüfung mit Medizinprodukten
Entfällt.

4 Auswahl der Patienten

4.1 Allgemeine Angaben
Es werden Patienten und Patientinnen der stationären kardiologischen, orthopädischen und psychosomatischen Rehabilitation in den Kooperationskliniken eingeschlossen, wenn sie die folgenden (vgl 4.2) Einschlusskriterien erfüllen und wenn kein Ausschlusskriterium (vgl. 4.3) vorliegt.

4.2 Einschlusskriterien
- Möglichkeit zum ungestörten Internetzugang;
- Alter 18 – 59 Jahre;
- Aktuelle Erwerbstätigkeit in Vollzeit, Teilzeit oder in Ausbildung;
- Deutliche berufliche Belastung im Screeningfragebogen;
- Deutsche Sprachkenntnisse

4.3 Ausschlusskriterien
- Arbeitslosigkeit, bewilligte Rente auf Zeit oder auf Dauer oder ausschließliche Tätigkeit als Hausfrau/ -mann ohne beabsichtigte Erwerbstätigkeit zu Rehabilitationsbeginn;
- Schwere der Erkrankung, so dass nicht an einer berufsbezogenen Schulungsgruppe teilgenommen werden kann (akute Bypass- bzw. Herzklappenoperation, schwere limitierende Begleiterkrankungen; akute schwere depressive Erkrankungen, hirnorganische Störungen, Suchterkrankungen oder schizophrene Erkrankungen)

4.4 Einsichtsfähigkeit
Es werden keine Patienten eingeschlossen, bei denen Zweifel an der Einwilligungsfähigkeit bestehen bzw. bei denen die Einsichtsfähigkeit nicht vorliegt.

4.5 Aufnahme in das Forschungsvorhaben
Die Patienten werden, wenn sie die Einschlusskriterien erfüllen, im Rahmen ihrer Maßnahme der stationären Rehabilitation nach ausführlicher Aufklärung und schriftlicher Erteilung des Einverständnisses in die Studie aufgenommen.

4.6 Ersetzen von Patienten
Nicht vorgesehen.

5 Ablauf
5.1 Angabe über die Einnahme der Prüfsubstanz
Entfällt.

5.2 Angabe über studienbedingte diagnostische und therapeutische Maßnahmen
Patienten der IG erhalten während der stationären Rehabilitation das „Gesundheitstraining Stressbewältigung am Arbeitsplatz – GSA“[2] und im Anschluss an die stationäre Rehabilitation das mit der stationären Behandlung vernetzte internetbasierte Nachsorgeangebot. Aufbauend auf das stationäre Modul erhält die IG drei Monate lang über die Plattform eine internetbasierte Nachsorgemaßnahme mit Arbeitsmaterialien, Entspannungsübungen und einer strukturierten Schreibaufgabe, im Sinne eines wöchentlichen Berichts, der durch den Online-Therapeuten kommentiert wird. Zum Ende der Intervention (= drei Monate poststationär) sowie insgesamt 12 Monate nach Entlassung aus der stationären Rehabilitation wird die IG nachbefragt.

5.3 Beschreibung der Behandlung der Kontrollgruppe
Patienten der KG erhalten ebenso das stationäre Angebot „GSA“ und werden über die bereits vorhandenen Möglichkeiten der ambulanten Reha-Nachsorge sowie Psychotherapie informiert („Treatment as usual“). Im Sinne eines Aufmerksamkeitsplacebos werden allen Teilnehmern der KG ab Ende der stationären Rehabilitation für die Dauer von drei Monaten regelmäßig über die Internetplattform Links zur Verfügung gestellt, die sie zu allgemein zugänglichen Informationen zur Nachsorge und Materialien zum Umgang mit beruflichen Belastungen führen. Ebenso werden sie poststationär drei und 12 Monate nachbefragt.

5.4 Angaben darüber, ob Vor- oder Begleitmedikation modifiziert oder abgesetzt werden soll
Entfällt.

5.5 Angaben zu speziellen Belastungstests
Entfällt.

5.6 Kontrolle des Gesundheitszustands der Patienten vor, während und nach dem Forschungsvorhaben
Eingeschlossen werden PatientInnen, die sich in stationärer medizinischer Rehabilitation befinden, so dass die Kontrolle des Gesundheitszustandes im Rahmen der Behandlungsmaßnahme geschieht. Im Rahmen der internetbasierten Nachsorgemaßnahme werden zu festen Befragungszeitpunkten der Gesundheitszustand und subjektive Beschwerden in standardisierter Form durch Selbsteinschätzungsverfahren erfasst, ebenso in den Follow-Up Befragungen.

5.7 Regeln zum Abbruch des Forschungsvorhabens für den Einzelnen wie auch für die Gesamtstudie
Studienabbruch des Einzelnen: Jeder Patient hat zu jedem Zeitpunkt das Recht, sein Einverständnis zur Durchführung der Studie ohne Angabe von Gründen zurückzuziehen. Die Studie wird ebenso für den Einzelnen abgebrochen, wenn eines der Ausschlusskriterien (vgl. 4.3) eintritt.
Die Gesamtstudie wird abgebrochen, wenn nicht hinreichend viele Patienten rekrutiert werden können. Die geplanten Befragungen stellen kein gesundheitliches Risiko für die Patienten dar, so dass ein Abbruch der Gesamtstudie aus diesem Grund nicht zu erwarten ist.

---
[2] http://www.zentrum-patientenschulung.de/datenbank/steckbrief.php?schulungen_id=113
http://forschung.deutsche-rentenversicherung.de/ForschPortalWeb/ressource?key=Manual_%20Gesundheitstraining_am_Arbeitsplatz_GSA.pdf

5.8 Regeln für die Fortführung einer Therapie nach Studienende

Eine Weiterbehandlung nach Abschluss der internetbasierten Nachsorgemaßnahme ist nicht vorgesehen.

6 Feststellung der Wirksamkeit

Primäre Zielgröße ist der „Sozialmedizinische Risikoindex" im Screening-Instrument SIBAR (Bürger & Deck, 2009) zum Zeitpunkt der Katamnese. Mit der o.g. Skala im SIBAR liegt ein umfassendes Maß zur Bestimmung beruflicher Problemlagen vor, das u.a. die subjektive Erwerbsprognose, die Dauer der Arbeitsunfähigkeit und Arbeitslosigkeit sowie ein vorliegendes Rentenbegehren abfragt und mit dem Summenscore eine gute prädiktive Validität für die spätere berufliche Reintegration besitzt.

Nebenzielgrößen sind Maße der beruflichen Belastung und berufsbezogene Einstellungen, erfasst über den Fragebogen zu *„Arbeitsbezogenen Verhaltens- und Erlebensmustern"* (AVEM, Schaarschmidt & Fischer, 1996), der *„Fragebogen zur Erfassung beruflicher Gratifikationskrisen"* (ERI, Siegrist, et al., 2004), die körperliche und psychische Funktionsfähigkeit erfasst über den *„Fragebogen zum Gesundheitszustand"* (SF-12, Bullinger & Kirchberger, 1998), Angst- und Depressivität erfasst über die deutsche Version der *„Hospital Anxiety and Depression Scale"* (HADS-D, Hermann, Buss, & Snaith, 1995), Symptombelastung, erfasst über den *„Patient Health Questionnaire"* (PHQ, Grafe, Zipfel, Herzog, & Lowe, 2004) und die Zufriedenheit mit der Rehabilitationsbehandlung, erfasst über den *„Zufriedenheitsfragebogen für Rehabilitationspatienten"* (ZUF-8, Schmidt, Lamprecht, & Wittmann, 1994).

7 Statistik und Auswertung

7.1 Auswertung der Prüfgrößen mit Darstellung der statistischen Methoden bei multi-zentrischen Studien

Die Evaluation erfolgt anhand von Gruppenvergleichen in der primären und den sekundären Zielgrößen drei und 12 Monate nach Entlassung mit den entsprechenden parametrischen und nonparametrischen statistischen Verfahren. Zur Beurteilung von Behandlungseffekten werden Effektstärken berechnet. Bei zu erwartenden kleinen Effekten (d = 0,3), einer Power von 0,80 und einem Alpha-Niveau von 0,05 ist eine Fallzahl von n = 310 pro Gruppe erforderlich. Bei einer angestrebten Stichprobe von insgesamt N = 800 Patienten, davon n = 400 Teilnehmer der IG (n = 100 je Kooperationsklinik) und n = 400 Teilnehmer der KG (n = 100 je Kooperationsklinik) ist diese Fallzahl auch bei einer erwarteten Dropout-Rate von 20% zu erreichen, so dass statistisch abgesicherte Aussagen getroffen werden können.

Die Erfassung der quantitativ erhobenen Daten erfolgt ebenso wie deren deskriptiv- und inferenzstatistische Auswertung mit Hilfe des Programmsystems PASW Statistics 18.

7.2 Prüfbögen

Prüfbögen nicht erforderlich.

7.3 Angaben über Zwischenauswertungen

Eine Zwischenauswertung findet nicht statt.

7.4 Angaben über den Umgang mit den Patientendaten

Alle Patienten (Interventions- und Kontrollgruppe) werden mündlich wie schriftlich über die Durchführung der Befragungen (einschließlich der Katamnese), die Zuweisung zu den Studiengruppen (Randomisierung) sowie über die Versuchsbedingungen aufgeklärt. Patienteninformationen mit schriftlicher Einwilligung werden entsprechend dem

Datenschutzkonzept der DRV-Bund den Patienten vorgelegt. Darin sind (1) als verantwortliche Stelle für das Forschungsvorhaben das Studienzentrum an der Universität Mainz und die jeweiligen Ansprechpartner in den Kooperationskliniken genannt. Weiterhin wird (2) das Forschungsvorhaben benannt und als solches von der eigentlichen Rehabilitationsbehandlung abgegrenzt gekennzeichnet, der (3) Ablauf des Projekts und die Aufgaben der Patienten werden in einer verständlichen Art und Weise beschrieben, ebenso wie (4) die Wahrung der Datenschutzrichtlinien beim Umgang mit den Forschungsdaten. Diesbezüglich werden die Patienten darüber aufgeklärt, dass die Auswertung der Daten in pseudonymisierter Form erfolgt, indem jedem Studienteilnehmer eine Studiennummer zugeordnet wird, unter der die erhobenen Daten gesammelt und an das Studienzentrum zur Weiterverarbeitung übermittelt und dort gespeichert werden. Eine getrennt davon geführte Liste, in der die Studiennummern den Teilnehmerdaten (Name und Kontaktdaten) zugeordnet sind sowie die Einwilligungserklärungen, sind nur einem unabhängigen Datentreuhänder des „Interdisziplinären Zentrums Klinische Studien“ in Mainz zugänglich, z.B. um die Patienten zur Nachbefragung zu kontaktieren. Für die spätere Datenauswertung werden nur Datensätze, die mit der Studiennummer codiert sind, verarbeitet. Die Zuordnungslisten werden nach Abschluss der Datenerhebung und die Forschungsdaten werden nach der üblichen Aufbewahrungszeit (10 Jahre) vernichtet. Weiterhin werden die Patienten (5) darüber informiert, dass ihre Teilnahme freiwillig ist, keine Nachteile durch Nichtteilnahme entstehen, eine gegebene Einwilligung jederzeit widerrufen werden kann (wobei personenbezogene Daten daraufhin umgehend und erhobene Forschungsdaten auf Wunsch gelöscht werden) und dass sie dies (6) durch ihre Unterschrift auf der Einverständniserklärung bestätigen müssen. Ebenso wird versichert, dass keine personenbezogenen Angaben über die Teilnahme am Forschungsprojekt an die Krankenkasse oder den Rentenversicherer weitergegeben werden.
Der Datenschutz und die Datensicherheit der Internetplattform werden durch eine im Internetbrowser automatisch per SSL (Secure Sockets Layer) verschlüsselte Verbindung, wie das vergleichsweise bei Banktransaktionen der Fall ist, die Verwendung von Pseudonymen seitens der Teilnehmer, so dass keine Rückschlüsse auf die reale Identität der Teilnehmer möglich ist, und einen durch eine Firewall abgesicherten Server gewährleistet.

8 Ethisch-rechtliche Aspekte

8.1 Gesetze, Vorschriften und Richtlinien

Die einschlägigen Paragraphen des AMG bzw. MPG (sowie entsprechende Ausführungsverordnungen), die letzte Fassung der Deklaration des Weltärztebundes über biomedizinische Forschung am Menschen, die Berufsordnung der deutschen Ärzte, das Medizinproduktegesetz und die entsprechenden Verordnungen, die Strahlenschutzverordnung und Röntgenverordnung, der Beschluss zur Überwachung der klinischen Prüfung von Arzneimitteln des Ausschusses Arzneimittel-, Apotheken- und Giftwesen, die Grundsätze für die ordnungsgemäße Durchführung der klinischen Prüfung von Arzneimitteln sowie die GCP-ICH-Richtlinien für klinische Prüfung von Arzneimitteln werden berücksichtigt.
Ebenso wird das gültige Datenschutzgesetz eingehalten (siehe auch 7.4).

8.2 Nutzen und Risiken

8.2.1 Nutzen

Der Nutzen für die an der Studie teilnehmenden PatientInnen besteht darin, dass sie in der Interventionsgruppe umgehend an die internetbasierte Nachsorgemaßnahme angebunden werden, die erzielten Verbesserungen der Bewältigungsmöglichkeiten im Umgang mit beruflichen Belastungen stabilisiert und weiter ausgebaut werden und hierdurch die berufliche Reintegration

gefördert wird. In der „Treatment as usual Gruppe“ besteht der Nutzen darin, dass die Patienten Informationen zu etablierten Therapiemöglichkeiten bei beruflichen Belastungen erhalten.

8.2.2 Risiken, Belastungen

Die stationären Therapieangebote werden durch einen/eine Assistenten/Assistentin der beteiligten Institutionen durchgeführt. Die Online-Intervention wird durch geschulte Psychotherapeutinnen und Psychotherapeuten durchgeführt. Hierdurch soll eine kompetente psychotherapeutische Unterstützung sichergestellt werden. Für den Fall, dass in der belastenden Phase des Übergangs vom stationären Angebot in den Alltag eine Krisensituation auftreten sollte, die sich nicht über das Medium Internet behandeln lässt, besteht die Möglichkeit eines 24-stündigen Notfall Telefonkontaktes, der durch erfahrene Psychotherapeuten der Klinik und Poliklinik für Psychosomatische Medizin und Psychotherapie der Universitätsmedizin Mainz entgegen genommen wird. Ist in diesem Rahmen keine ausreichende Unterstützung möglich, so wird der vom Patienten angegebene Kontakt zur Krisenintervention vor Ort eingeschaltet.

8.2.3 Zusätzliche Belastungen

Keine.

8.3 Komplikationen

siehe oben

8.4 Vorteile für Patienten

Siehe 8.2.1.

8.5 Versicherung

Es wird keine gesonderte Versicherung benötigt, da es sich nicht um eine Arzneimittel- oder Medizinproduktstudie handelt.

8.6 Ist der Prüfungsleiter entsprechend AMG und GCP-ICH Richtlinien informiert?

Ja

8.7 Wurden ähnliche Forschungsvorhaben oder vergleichbare Untersuchungen bereits durchgeführt? Wenn ja, mit welchem Ergebnis?

Keine

8.8 Aufklärung

Alle Patienten und Probanden werden entsprechend beiliegender Einverständniserklärung schriftlich informiert. Darüber hinaus erfolgt eine mündliche Aufklärung, im Rahmen derer auch Fragen gestellt und geklärt werden können. Es wird besonderer Wert auf eine allgemein verständliche Form gelegt, mit der über Wesen, Ablauf und Ziel des Forschungsvorhabens und damit verbundenen Maßnahmen aufgeklärt wurde. Auch über die Möglichkeit, zu jedem Zeitpunkt die weitere Teilnahme an der Studie ablehnen zu können, wird explizit informiert.

8.9 Einwilligungserklärung

Bitte entnehmen Sie den Text der schriftlichen Probandeninformation und Einwilligungserklärung dem Anhang.

8.10 Einsichtsfähigkeit, Einwilligungsfähigkeit

Es werden nur Patienten und Probanden in die Studie eingeschlossen, die geschäftsfähig sind und bei denen sowohl Einsichtsfähigkeit als auch Einwilligungsfähigkeit bestehen.

8.11 Verschiedenes

8.11.1 Ein Probanden-/Patientenausweis wird nicht ausgestellt

8.11.2 Information weiterbehandelnder Ärzte

Eine Benachrichtigung der Hausärzte von Probanden erscheint nicht notwendig, kann aber auf Wunsch des Probanden erfolgen.

8.11.3 Studienleiter

Die Studienleiter verfügen alle über die notwendige fachliche und persönliche Qualifikation zur Durchführung des Forschungsvorhabens:

- *Dipl.-Psych. Dr. Rüdiger Zwerenz* (Studienleiter) hat als wissenschaftlicher Mitarbeiter schon mehrere Drittmittelprojekte bearbeitet und eingeworben.
- *Prof. Manfred E. Beutel* ist Arzt für Psychosomatische Medizin und Psychotherapie Medizin und Direktor der Klinik und Poliklinik für Psychosomatische Medizin und Psychotherapie.
- *Jens Edinger* ist Prüfarzt und stellvertretender Leiter des „Studienzentrums Psychische Erkrankungen" (BMBF gefördert), einer Untereinheit des „Interdisziplinären Zentrums Klinische Studien" in Mainz.

Unterschriften

Dipl.-Psych. Dr. Rüdiger Zwerenz	Jens Edinger	Univ. Prof. Dr. Manfred E. Beutel
Studienleiter	Co-Studienleiter	Direktor der Klinik und Poliklinik für Psychosomatische Medizin und Psychotherapie

Briefkopf Kooperationsklinik

UNIVERSITÄTSmedizin. MAINZ

Klinik und Poliklinik für Psychosomatische Medizin
und Psychotherapie – Direktor: Univ.-Prof. Dr. M.E. Beutel

Einwilligungserklärung für Patienten

Studientitel: Wirksamkeit von internetbasierter Nachsorge in der medizinischen Rehabilitation

....

Name *Vorname*

....

Geburtsdatum

1. Ich bin in einem persönlichen Gespräch ausführlich und verständlich über Wesen, Bedeutung, Risiken und Tragweite der Studie aufgeklärt worden. Ich hatte die Gelegenheit zu einem Beratungsgespräch. Alle meine Fragen wurden zufriedenstellend beantwortet, ich kann jederzeit neue Fragen stellen. Ich habe darüber hinaus den Text der Patienteninformation gelesen und verstanden. Ich hatte ausreichend Zeit, mich zu entscheiden.
2. Ich verstehe, dass meine Teilnahme völlig freiwillig ist und dass ich jederzeit die Möglichkeit habe, meine Einwilligung ohne Angabe von Gründen zurückzuziehen (mündlich oder schriftlich), und dass dies keinerlei Einfluss auf meine medizinische Versorgung oder Rechte hat. Ferner habe ich zur Kenntnis genommen, dass die personenbezogenen Daten umgehend und die bis dahin erhobenen Forschungsdaten auf meinen Wunsch gelöscht werden.
3. Ich stimme zu, an der oben genannten Studie mitzuarbeiten, indem ich (a) einige Fragebögen ausfülle und (b) in eine der Studienbehandlungsgruppen zufällig eingeteilt werde und ggf. im Anschluss an die stationäre Rehabilitation an einer internetbasierten Nachsorgemaßnahme teilnehmen werde.
4. Mir wurde versichert, dass alle gesammelten Daten vertraulich behandelt und nur für die Zwecke des Forschungsvorhabens genutzt werden. **Ich habe verstanden und bin damit einverstanden, dass meine studienbezogenen Daten pseudonymisiert (d.h. kodiert ohne Angabe von Namen, Anschrift, Initialen oder Ähnliches) erhoben, auf Datenträgern gespeichert und vom Auftraggeber der Studie ausgewertet werden. Die Weitergabe an Dritte einschließlich Publikation erfolgt ausschließlich in anonymisierter Form, d.h. kann nicht meiner Person zugeordnet werden.**

5. Ich bin damit einverstanden, dass für den Zeitraum der Studiendurchführung von den Forschern eine Liste mit Namen und Adressen einerseits und dem mir zugeordneten Code andererseits geführt wird, damit mir für die Nachbefragung gegebenenfalls Fragebögen zugeschickt werden können. Diese Liste sowie die Einwilligungs-erklärungen sind während der Datenerhebungsphase nur einem unabhängigen Datentreuhänder des „Interdisziplinären Zentrums Klinische Studien“ in Mainz zugänglich.
6. Ich erkläre mich damit einverstanden, dass medizinische Befunde aus der klinischen Dokumentation der behandelnden Kliniken übernommen werden und entbinde die zuständigen MitarbeiterInnen diesbezüglich von der Schweigepflicht.
7. Ich bin bereit, nach Ende der stationären Behandlung, ggf. auch nach der internetbasierten Nachsorgemaßnahme per E-Mail, telefonisch oder Post von den Forschern bis spätestens 12 Monate nach Entlassung aus der Klinik kontaktiert zu werden.
8. Ein Exemplar der Patienteninformation und Einwilligungserklärung habe ich erhalten, gelesen und verstanden.

Ich bin einverstanden und nehme an der Studie teil.

....

Ort und Datum *Unterschrift des Studienteilnehmers*

....

Name des aufklärenden Studienmitarbeiters (in Druckbuchstaben)

Ich habe das Aufklärungsgespräch geführt und die Einwilligung des Teilnehmers eingeholt.

....

Ort und Datum *Unterschrift des Studienmitarbeiters*

Briefkopf Kooperationsklinik

UNIVERSITÄTSmedizin. MAINZ

Klinik und Poliklinik für Psychosomatische Medizin
und Psychotherapie – Direktor: Univ.-Prof. Dr. M.E. Beutel

Patienteninformation zur Studie

Wirksamkeit von internetbasierter Nachsorge in der medizinischen Rehabilitation

Sehr geehrte(r) Patient(in),

Sie befinden sich in ***Name der Kooperationsklinik*** zur stationären medizinischen Rehabilitation. Sie erhalten hier alle notwendigen und geeigneten Behandlungen, die zur wesentlichen Besserung Ihrer Gesundheit und Wiederherstellung Ihrer Leistungsfähigkeit beitragen. Darüber hinaus wollen wir in Zusammenarbeit mit der Universitätsmedizin Mainz (Klinik für Psychosomatische Medizin und Psychotherapie; Prof. Dr. M.E. Beutel) die im nachfolgenden beschriebene Studie durchführen.

Im heutigen Gesundheitswesen wird es immer wichtiger, die Ergebnisse durchgeführter Behandlungen ausreichend zu dokumentieren und in ihrem Verlauf zu untersuchen. Dies dient u.a. dazu, die tägliche Arbeit, die Behandlungskonzepte und Behandlungsformen zu überprüfen und ständig zu verbessern.

Wir wissen, dass auch nach erfolgreicher Rehabilitation die Rückkehr an den Arbeitsplatz oft neue Anforderungen oder Belastungen aufwirft. Aus diesem Grund führen wir eine wissenschaftliche Studie über die Wirkung von Schulungsprogrammen für Patienten mit beruflichen Belastungen durch. Es soll geprüft werden, inwiefern das Thema Beruf in die medizinische Rehabilitationsbehandlung integriert und dadurch die Behandlungsergebnisse verbessert werden können. Dazu haben wir ein spezielles Schulungsprogramm mit ggf. anschließender internetbasierter Nachsorgemaßnahme entwickelt. Sollten Sie die Bedingungen zur Studienteilnahme erfüllen, können wir Ihnen im Rahmen der Studie dieses Schulungsprogramm zusätzlich zur Standardtherapie anbieten und werden Sie per Los zufällig einer von zwei Studiengruppen zuweisen. Beide Gruppen erhalten Zugang zu einer durch das Studienzentrum der Universitätsmedizin Mainz verwalteten Internetplattform. Patienten der ersten Studiengruppe werden nach Abschluss der stationären Therapie in einer internetbasierten Nachsorgemaßnahme weiter betreut. Im Rahmen der internetbasierten Nachsorgemaßnahme bekommen Sie über die Internetplattform für einen Zeitraum von drei Monaten eine regelmäßige therapeutische Weiterbetreuung, u.a. mit Arbeitsmaterialien, Entspannungsübungen und therapeutischen Aufgaben mit direkter Rückmeldung. Für die Teilnahme an der internetbasierten Nachsorge wird ein Zeitaufwand von ca. 60 Minuten pro Woche nötig sein. Patienten der zweiten Studiengruppe bekommen nach der stationären Rehabilitationsmaßnahme über die Internetplattform regelmäßig Links mit allgemein zugänglichen Informationen zur Nachsorge sowie Materialien und Hilfen zum Umgang mit beruflichen Belastungen zur Verfügung gestellt. Zur Nutzung der Informationen wird ein vergleichbarer wöchentlicher Zeitaufwand von ca. 60 Minuten nötig sein.

Ziel der Studie wird es sein, die Wirksamkeit der internetbasierten Nachsorgemaßnahme zu überprüfen. Deshalb möchten wir Sie ergänzend zu den Behandlungen bitten, Fragebögen auszufüllen, die sich auf Ihren Gesundheitszustand, Ihre Erkrankung, Ihre soziale und berufliche Situation sowie Ihre Zufriedenheit mit der Behandlung in unserer Klinik beziehen. Zusätzlich möchten wir medizinische Befunde aus der klinischen Dokumentation der behandelnden Kliniken übernehmen. Die Weitergabe der medizinischen Befunde aus den behandelnden Kliniken an das Forscherteam kann nur

erfolgen, wenn die damit betrauten MitarbeiterInnen der Klinik von der Schweigepflicht entbunden sind.

Ebenso möchten wir Sie bitten, drei Monate sowie ein Jahr nach Entlassung Fragebögen auszufüllen, die sich auf Ihren Gesundheitszustand und Ihre Erfahrungen und eventuelle Probleme bei Ihrer Rückkehr an den Arbeitsplatz beziehen. Die Fragebögen werden für Sie zu den entsprechenden Zeitpunkten auf der Internetplattform hinterlegt sein. Alternativ können Sie die Fragebögen auch zugesandt bekommen, die Sie bitte ausfüllen und im beigefügten Freiumschlag an das Studienzentrum zurücksenden. Für diese Befragung müssen sie jeweils ca. 45 Minuten Bearbeitungszeit einplanen.

Sie können sich ohne Einschränkung darauf verlassen, dass alle Ihre Angaben absolut vertraulich behandelt werden. Alle Daten werden durch die Forschungsmitarbeiter pseudonymisiert (d.h. kodiert ohne Angabe von Namen, Anschrift, Initialen oder Ähnliches) erhoben, auf Datenträgern gespeichert und vom Auftraggeber der Studie ausgewertet werden. Zu Teilnahmebeginn wird dafür jedem Patienten ein Code zugeordnet, unter welchem dann alle Fragebögen und Daten pseudonymisiert an das Forschungsteam weitergeleitet werden. Damit Ihnen z.B. bei Befragung per Post ein Jahr nach Entlassung ein Fragebogen von den Forschern zugeschickt werden kann, ist es notwendig, dass eine Liste mit Namen und Adressen einerseits und dem Code der teilnehmenden Patienten andererseits geführt wird. Diese Liste sowie die Einwilligungs-erklärungen sind während der Datenerhebungsphase nur einem unabhängigen Datentreuhänder des „Interdisziplinären Zentrums Klinische Studien“ in Mainz zugänglich. Nach Abschluss der Studie wird diese Liste zusammen mit den anderen Unterlagen, die Personendaten enthalten, vernichtet werden.

Die Internetplattform ist nach den aktuellen Sicherheitsstandards abgesichert, eine Zuordnung personenbezogener Daten ist nicht möglich, da die Daten mit direktem Personenbezug (siehe oben) nicht online erhoben oder gespeichert sind und Sie sich mit einem anonymen Kennzeichen anmelden. Bei Anmeldung auf der Internetplattform werden Sie gebeten eine E-Mail-Adresse anzugeben, über die Sie automatisch benachrichtigt werden, wenn Informationen, therapeutische Rückmeldungen oder auszufüllende Fragebögen für Sie hinterlegt sind. Sollten Sie keine E-Mail-Adresse angeben, besteht die Möglichkeit, die Benachrichtigungen telefonisch zu erhalten.

Alle statistischen Auswertungen erfolgen pseudonymisiert, d.h. ohne Angaben, die Rückschlüsse auf Ihre Person zulassen würden. Bei eventuellen Veröffentlichungen, z.B. in Fachzeitschriften, wird selbstverständlich kein Bezug zu Ihrer Person hergestellt. Es werden im Zusammenhang mit der Studie keine personenbezogenen Daten an Dritte – auch nicht an Rentenversicherungsträger, Krankenkasse oder andere Institutionen – weitergegeben. Die in dieser Studie erhobenen Daten werden gelöscht, sobald sie für die weitere wissenschaftliche Auswertung nicht mehr erforderlich sind.

Die Teilnahme an der Studie ist freiwillig. Die Studie wird durch die Klinik und Poliklinik für Psychosomatische Medizin und Psychotherapie der Universitätsmedizin Mainz im Interesse einer besseren Patientenversorgung durchgeführt und wurde nicht von Kostenträgern (z.B. DRV oder Krankenkasse) oder sonstigen Dritten veranlasst. Sie können die Teilnahme jederzeit ohne Angabe von Gründen – auch bei schon gegebener Einwilligung – beenden. Personenbezogene Daten werden daraufhin umgehend gelöscht, auf Ihren Wunsch ebenso bereits erhobene Forschungsdaten. Auch wenn Sie die Teilnahme ablehnen, erwachsen Ihnen hieraus keine Nachteile. Wir möchten Sie bitten, alle Fragen offen und ehrlich zu beantworten – für uns gibt es keine „erwünschten“ oder „nicht erwünschten“ Antworten!

Wir danken Ihnen für Ihr Verständnis und bitten Sie freundlich darum, an der Studie teilzunehmen. Um unsere Behandlung zu verbessern, sind wir auf die Mithilfe unserer Patienten angewiesen. Ihre Angaben sind eine wichtige Rückmeldung für uns.

Für Ihre Unterstützung und Mitarbeit möchten wir uns im Voraus bei Ihnen bedanken!

Dr. NN	Prof. Dr. Manfred Beutel	Dr. Rüdiger Zwerenz
Kooperationsklinik	Studienzentrum Mainz	Studienzentrum Mainz
Leiter der Kooperationsklinik	*Leiter der Klinik für Psychosomatische Medizin und Psychotherapie*	*Verantwortlicher Studienleiter*

Literaturverzeichnis

BAR (2002). *Berufsbezogene Maßnahmen in der medizinischen Rehabilitation - bisherige Entwicklungen und aktuelle Perspektiven*. Frankfurt: BAR.

Bee, P. E., Bower, P., Lovell, K., Gilbody, S., Richards, D., Gask, L., et al. (2008). Psychotherapy mediated by remote communication technologies: a meta-analytic review. *BMC Psychiatry, 8*(60).

Beutel, M. E., Dommer, T., Kayser, E., Bleichner, F., Vorndran, A., & Schlüter, K. (1999). Arbeit und berufliche Integration psychosomatisch Kranker. Nutzen und Indikation der beruflichen Belastungserprobung. *Psychotherapie, Psychosomatik, medizinische Psychologie, 49*(9/10), 368-374.

Beutel, M. E., Gerhard, C., Kayser, E., Gustson, D., Weiss, B., & Bleichner, F. (2002). Berufsbezogene Therapiegruppen für ältere Arbeitnehmer im Rahmen der tiefenpsychologisch orientierten psychosomatischen Rehabilitation. *Gruppenpsychotherapie und Gruppendynamik, 38*(4), 313-334.

Beutel, M. E., Gerhard, C., Wagner, S., Bittner, R., Bleichner, F., Schattenburg, L., et al. (2004). Verminderung von Technologieängsten in der psychosomatischen Rehabilitation - Konzepte und Ergebnisse zu einem Computertraining für ältere Arbeitnehmer. *Zeitschrift für Gerontologie und Geriatrie, 37*, 221-230.

Beutel, M. E., Knickenberg, R. J., Krug, B., Mund, S., Schattenburg, L., & Zwerenz, R. (2006). Psychodynamic focal group treatment for psychosomatic inpatients - with an emphasis on work-related conflicts. *International Journal for Group Psychotherapy, 56*(3), 285-306.

Beutel, M. E., Zwerenz , R., Bleichner, F., Vorndran, A., Gustson, D., & Knickenberg, R. J. (2005). Vocational training integrated into inpatient psychosomatic rehabilitation - Short and long-term results from a controlled study. *Disability & Rehabilitation, 27*(15), 891-900.

Beutel, M. E., Zwerenz, R., Kayser, E., Schattenburg, L., & Knickenberg, R. J. (2004). Berufsbezogene Einstellungen, Ressourcen und Risikomerkmale im Therapieverlauf: Eignet sich der AVEM als Messverfahren für psychisch und psychosomatisch Kranke? *Zeitschrift für Klinische Psychologie und Psychotherapie, 33*(2), 110-119.

BfA (2000). *Eckpunkte arbeitsbezogener Strategien in der medizinischen Rehabilitation*.Unpublished manuscript, Berlin.

Bullinger, M., & Kirchberger, I. (1998). *SF-36 Fragebogen zum Gesundheitszustand. Handanweisung*. Göttingen: Hogrefe.

Bürger, W., & Deck, R. (2009). SIBAR - ein kurzes Screening-Instrument zur Messung des Bedarfs an berufsbezogenen Behandlungsangeboten in der medizinischen Rehabilitation. *Rehabilitation, 48*, 211-221.

DAK (2010). *DAK Gesundheitsreport 2010*. Hamburg: DAK.

Fiedler, R. G., Ranft, A., Greitemann, B., & Heuft, G. (2005). Arbeitsmotivation - Diagnostikinstrumente und ihre Relevanz in der Patientenversorgung. Zum Stand arbeitsbezogener Motivationsdiagnostik. *Psychotherapie, Psychosomatik, medizinische Psychologie, 55*, 469-475.

Frese, M. (1991). Streßbedingungen in der Arbeit und psychosomatische Beschwerden: eine kausale Interpretation. In S. Greif, E. Bamberg & N. Semmer (Eds.), *Psychischer Streß am Arbeitsplatz* (pp. 120-134). Göttingen: Hogrefe.

Golkaramnay, V., Cicholas, B., & Vogler, J. (2010). Wirksamkeit einer internetgestützten Nachsorge nach stationärer psychosomatischer Rehabilitation. In Deutsche Rentenversicherung Bund (Ed.), *19. Rehabilitationswissenschaftliches Kolloquium:*

Innovation in der Rehabilitation - Kommunikation und Vernetzung vom 8. bis 10. März 2010 in Leipzig (pp. 171-172). Berlin: Deutsche Rentenversicherung Bund,.

Grafe, K., Zipfel, S., Herzog, W., & Lowe, B. (2004). Screening for psychiatric disorders with the Patient Health Questionnaire (PHQ). Results from the German validation study. *Diagnostica, 50*(4), 171-181.

Hermann, C., Buss, U., & Snaith, R. (1995). *HADS-D - Hospital Anxiety and Depression Scale - Deutsche Version: Ein Fragebogen zur Erfassung von Angst und Depressivität in der somatischen Medizin, Testdokumentation und Handanweisung*. Bern: Huber.

Hillert, A., Koch, S., Beutel, M. E., Holme, M., Knickenberg, R., Middeldorf, S., et al. (2007). Berufliche Belastungen und Indikationsstellung für ein berufsbezogenes Schulungsmodul in der medizinischen Rehabilitation: Bericht einer multizentrischen Evaluationsstudie. *Praxis Klinische Verhaltensmedizin und Rehabilitation, 77*, 147-154.

Irle, H., Klosterhuis, H., & Grünbeck, P. (1998). Sozialmedizinische Prognose nach stationärer medizinischer Rehabilitation in der Angestelltenversicherung. *Praxis Klinische Verhaltensmedizin und Rehabilitation*(42), 51-60.

Kersting, A., Schlicht, S., & Kroker, K. (2009). Internettherapie. Möglichkeiten und Grenzen. *Nervenarzt, 80*, 797-804.

Koch, S., Hedlund, S., Rosenthal, S., & Hillert, A. (2006). Stressbewältigung am Arbeitsplatz: Ein stationäres Gruppentherapieprogramm. *Verhaltenstherapie, 16*, 7-15.

Koch, U., Bürger, W., Schulz, H., Glier, B., & Rodewig, K. (1997). Berufbezogene Behandlungsangebote in der psychosomatischen Rehabilitation: Bedarf und Konzeption. *Deutsche Rentenversicherung, 9-10*, 548-574.

Löffler, S., Wolf, H.-D., & Vogel, H. (2008). Das Würzburger Screening zur Identifikation von beruflichen Problemlagen. In Deutsche Rentenversicherung Bund (Ed.), *Evidenzbasierte Rehabilitation - zwischen Standardisierung und Individualisierung. 17. Rehabilitationswissenschaftliches Kolloquium vom 3. bis 5. März in Bremen* (Vol. 77, pp. 217-219). Berlin: wdv.

Müller-Fahrnow, W., Greitemann, B., Radoschewski, F., Gerwinn, H., & Hansmeier, T. (2005). Berufliche Orientierung in der medizinischen Rehabilitation und Leistungen zur Teilhabe am Arbeitsleben. *Rehabilitation, 44*, 287-296.

Rudolf, S., & Heitzmann, B. (2006). "Fit für den Beruf". Entwicklung und Evaluation eines AVEM-gestützten Patientenschulungsprogramms zur beruflichen Orientierung für die stationäre Rehabilitation. In Deutsche Rentenversicherung Bund (Ed.), *15. Rehabilitationswissenschaftliches Kolloquium. Rehabilitation und Arbeitswelt - Herausforderungen und Strategien - vom 13. bis 15. März 2006 in Bayreuth* (Vol. 64, pp. 76-78). Bad Homburg wdv.

Schaarschmidt, U., & Fischer, A. (1996). *Arbeitsbezogenes Verhaltens- und Erlebensmuster*. Frankfurt: Swets & Zeitlinger.

Schmidt, J., Lamprecht, F., & Wittmann, W. W. (1994). Zufriedenheit mit der stationären Versorgung. Entwicklung eines Fragebogens und erste Validitätsuntersuchungen. *Psychotherapie, Medizinische Psychologie, 39*, 248-255.

Siegrist, J. (2002). Stress am Arbeitsplatz. In R. Schwarzer, M. Jerusalem & H. Weber (Eds.), *Gesundheitspsychologie von A bis Z. Ein Handwörterbuch* (pp. 554-557). Göttingen: Hogrefe.

Siegrist, J., Starke, D., Chandola, T., Godin, I., Marmot, M., Niedhammer, I., et al. (2004). The measurement of effort-reward imbalance at work: European comparisons. *Social Science And Medicine, 58*, 1483-1499.

Streibelt, M. (2009). Validität und Reliabilität eines Screening-Instruments zur Erkennung besonderer beruflicher Problemlagen bei chronischen Krankheiten (SIMBO-C). *Rehabilitation, 48*, 135-144.

Theissing, J., & Deck, R. (2009). Reha-Nachsorge per Internet: Akzeptanz und Kompetenzen bei Patienten mit abdomineller Adipositas in der kardio-diabetologischen Rehabilitation. In Deutsche Rentenversicherung Bund (Ed.), *18. Rehabilitationswissenschaftliches Kolloquium. Innovation in der Rehabilitation - Kommunikation und Vernetzung, vom 09. bis 11. März 2009 in Münster* (pp. 44-46). Berlin: wdv.

VDR (1992). *Bericht der Reha-Kommission des Verbandes Deutscher Rentenversicherungsträger. Empfehlungen zur Weiterentwicklung der medizinischen Rehabilitation in der gesetzlichen Rentenversicherung*. Frankfurt/Main: VDR.

Wagner, S., Kaschel, R., Paulsen, S., Bleichner, F., Knickenberg, R. J., & Beutel, M. (2008). Does a cognitive training programme improve the performance of middle-aged employees undergoing in-patient psychosomatic treatment? *Disability and Rehabilitation, 30*, 1786-1793.

Wagner, S., Kaschel, R., Paulsen, S., Knickenberg, R., Bleichner, F., & Beutel, M. (2006). Kognitive Auffälligkeiten, Depressivität und Leistungsfähigkeit bei älteren Arbeitnehmern in stationärer psychosomatischer Behandlung. *Der Nervenarzt, 77*(11), 1338-1344.

Winkelhake, U., Schutzeichel, F., Niemann, O., & Daalmann, H. (2003). Die berufsorientierte medizinische Rehabilitation (BOR) bei Funktionseinschränkungen aufgrund orthopädischer Erkrankungen. *Rehabilitation, 42*, 30-35.

Zwerenz, R., Knickenberg, R. J., Schattenburg, L., & Beutel, M. E. (2004). Berufliche Belastungen und Ressourcen berufstätiger Patienten der psychosomatischen Rehabilitation im Vergleich zur Allgemeinbevölkerung. *Rehabilitation, 43*(1), 10-16.

Zwerenz, R., Knickenberg, R. J., Schattenburg, L., & Beutel, M. E. (2005). Motivation zur psychosomatisch-psychotherapeutischen Bearbeitung von beruflichen Belastungen - Entwicklung und Validierung eines Fragebogens. *Rehabilitation, 44*, 14-23.
